# Single-Cell Transcriptomics Revealed Subtype-Specific Tumor Immune Microenvironments in Human Glioblastomas

**DOI:** 10.3389/fimmu.2022.914236

**Published:** 2022-05-20

**Authors:** Yong Xiao, Zhen Wang, Mengjie Zhao, Yanxiang Deng, Mingyu Yang, Graham Su, Kun Yang, Chunfa Qian, Xinhua Hu, Yong Liu, Liangyuan Geng, Yang Xiao, Yuanjie Zou, Xianglong Tang, Hongyi Liu, Hong Xiao, Rong Fan

**Affiliations:** ^1^ Department of Biomedical Engineering, Yale University, New Haven, CT, United States; ^2^ Department of Neurosurgery, Nanjing Brain Hospital Affiliated to Nanjing Medical University, Nanjing, China; ^3^ Department of Neuro-Psychiatric Institute, Nanjing Brain Hospital Affiliated to Nanjing Medical University, Nanjing, China; ^4^ Yale Stem Cell Center and Yale Cancer Center, Yale School of Medicine, New Haven, CT, United States; ^5^ Human and Translational Immunology Program, Yale School of Medicine, New Haven, CT, United States

**Keywords:** single-cell RNA sequencing, glioblastoma, cellular state, tumor-associated macrophage, hypoxia, M2-type polarization, cell-to-cell interaction

## Abstract

Human glioblastoma (GBM), the most aggressive brain tumor, comprises six major subtypes of malignant cells, giving rise to both inter-patient and intra-tumor heterogeneity. The interaction between different tumor subtypes and non-malignant cells to collectively shape a tumor microenvironment has not been systematically characterized. Herein, we sampled the cellular milieu of surgically resected primary tumors from 7 GBM patients using single-cell transcriptome sequencing. A lineage relationship analysis revealed that a neural-progenitor-2-like (NPC2-like) state with high metabolic activity was associated with the tumor cells of origin. Mesenchymal-1-like (MES1-like) and mesenchymal-2-like (MES2-like) tumor cells correlated strongly with immune infiltration and chronic hypoxia niche responses. We identified four subsets of tumor-associated macrophages/microglia (TAMs), among which TAM-1 co-opted both acute and chronic hypoxia-response signatures, implicated in tumor angiogenesis, invasion, and poor prognosis. MES-like GBM cells expressed the highest number of M2-promoting ligands compared to other cellular states while all six states were associated with TAM M2-type polarization and immunosuppression *via* a set of 10 ligand–receptor signaling pathways. Our results provide new insights into the differential roles of GBM cell subtypes in the tumor immune microenvironment that may be deployed for patient stratification and personalized treatment.

## Introduction

Isocitrate dehydrogenase (IDH)-wild-type glioblastoma (GBM) is an incurable brain tumor, and the main underlying challenge to treatment is heterogeneity (1). At least three determinants drive GBM heterogeneity: (i) genetic alterations reshape cellular transformation, which induces tumorigenesis; (ii) cellular lineages and the epigenetic programs contribute to key phenotype; and (iii) the tumor microenvironment (TME) (2). Although GBM differs in individuals, investigations have attempted to uncover the common ground shared among most patients, in hopes of providing new insights into treatment. In the bulk sequencing era, The Cancer Genome Atlas (TCGA) Research Network generated a blueprint of GBM genomic subtypes, namely, classical, mesenchymal, neural, and proneural subtypes each having a unique signature (3). However, multiple TCGA subtypes can co-exist in the same tumor of the same patient either in different regions or even in close proximity, and these subtypes can change over time and evolve through treatment as seen by longitudinal genomic analysis (4). The advent of single-cell RNA sequencing (scRNA-seq) provides an opportunity to dissect the lineage identity and heterogeneity of cancers with unprecedented resolution. Neftel et al. used scRNA-seq to examine GBM tumor cells and found that the malignant cells share a limited set of cellular states, namely, astrocyte-like (AC-like), mesenchymal-1-like (MES1-like), mesenchymal-2-like (MES2-like), oligodendrocyte-progenitor-like (OPC-like), neural-progenitor-1-like (NPC1-like), and neural-progenitor-2-like (NPC2-like) states (5). Moreover, these cellular states are partially enriched for select genetic events: amplifications of *EGFR*, *PDGFRA*, and *CDK4* are more common in AC-like, OPC-like, and NPC-like states, respectively, whereas mutations of *NF1* are more common in MES-like states. These works provide a basis for studying the heterogeneity of GBM malignant cells, but the specific characteristics of different cellular states and their roles in shaping the tumor immune microenvironment and subsequently patient outcomes need to be systematically studied. The interactions between different tumor cellular states and non-malignant cells (e.g., vascular and immune cells) are yet to be elucidated in order to gain a holistic view of the TME in GBM patients.

TME is composed of malignant tumor cells together with surrounding non-malignant stromal cells including vascular and immune cells as well as non-cellular components such as the extracellular matrix. These cell types communicate with each other *via* ligand–receptor interactions, which play crucial roles in inflammation, immune infiltration, tumorigenesis, and therapeutic resistance (6). Although scRNA-seq has emerged as a powerful method to dissect cellular states within tumors and to study the cross-talk between cells (7), in the field of human GBM research, scRNA-seq studies were mostly concentrated on quantitating the heterogeneity of malignant tumor cells or tumor stem/progenitor cells (5, 8, 9). In glioma, stromal cells comprise normal astrocytes, oligodendrocytes, immune cells, and endothelial cells (5, 10). Lines of evidence from experimental and clinical studies have shown that tumor-associated macrophages/microglia (TAMs) make up most of the immune cells in GBM (>95%) (11–13), but we have a limited understanding of the heterogeneity of GBM TAMs and the subtypes of TAMs contributing to GBM patient bleak prognosis (14, 15). TAMs have been functionally divided into M1 and M2 polarized cells, and the latter is associated with tumor cell invasion, angiogenesis, and suppressive antitumor immunity, resulting in poor prognosis (16–18). Yuan et al. and Zhang et al. used the same published dataset to examine the interactions between glioma tumor cells and TAMs (19, 20), but have yet to investigate the differential roles of the GBM subtypes and these interactions in TAM M2-type polarization. Although some studies indicate that glioma cells may recruit TAMs through the generation of soluble factors, such as *CSF*, *MCP*, *CX3CL1*, *CCL2*, and *EGF* (21), the contributions of major regulatory pathways and their modulators or targets involved in TAM polarization are inadequately studied. Thus, researchers have yet to systematically examine the role of six GBM cellular states in cell–cell communication and TAM polarization in order to elucidate the mechanisms underlying TAM polarization to discover new strategies for treating glioma by intervening cell–cell interactions. 

Here, we report on the scRNA-seq of primary IDH-wild-type tumors surgically resected from 7 GBM patients and obtained 28,279 single-cell transcriptomes. We dissociated the tumor specimens immediately after procurement at the operating room to prepare samples for scRNA-seq without cell sorting with *CD45* antibody conducted in previous studies (5, 22) and therefore all major cell types in the GBM samples including tumor cells and stromal cells were retained and analyzed in our data, which enabled us to explore important questions such as which tumor cellular states could be associated with GBM progenitor cells, which cell types were poor-prognosis indicators, how GBM tumor cells reprogram TAMs into an immunosuppressive phenotype, and how they communicate with other stromal cells to shape the subtype-specific TME. We found that NPC2-like tumor cells functioned as tumor cells of origin, and that hypoxia-response MES-like tumor cells and hypoxia-response TAMs were involved in angiogenesis and the invasion niche development. Additionally, our work provided the first systematic study of the landscape of cell–cell interaction and gene regulation network in shaping the GBM microenvironments including promoting TAM M2-type polarization, endothelial angiogenesis, and their relationship with different GBM cellular states, which may shed new light to the development of therapeutic approaches by targeting TME components.

## Methods

### Tumor Tissue Acquisition and Processing

Fresh tumor samples were acquired when patients underwent surgical resection of primary GBM. Sample use was approved by the Institutional Review Board at the Nanjing Brain Hospital Affiliated to Nanjing Medical University. The experiments performed here conform to the principles set out in the WMA Declaration of Helsinki and the Department of Health and Human Services Belmont report. All patients signed informed consent. Their pathological results were confirmed as IDH-wild-type GBM according to the WHO 2016 Classification. Fresh tumor samples were immediately stored in the GEXSCOPE Tissue Preservation Solution (Singleron Biotechnologies) at 2–8°C after resection. Prior to tissue dissociation, the specimens were washed three times with Hanks’ Balanced Salt Solution (HBSS) and minced into 1- to 2-mm pieces. Subsequently, these pieces were digested in 2 ml of GEXSCOPE Tissue Dissociation Solution (Singleron Biotechnologies) at 37°C for 15 min in a 15-ml centrifuge tube with continuous agitation. Following digestion, a 40-micron sterile strainer (Corning) was used to separate cells from cell debris and other impurities. Then, cells were centrifuged at 1,000 rpm, 4°C, for 5 min and cell pellets were resuspended into 1 ml of PBS (HyClone). To remove red blood cells, 2 ml of GEXSCOPE Red Blood Cell Lysis Buffer (Singleron Biotechnologies) was added to the cell suspension and incubated at 25°C for 10 min. The mixture was then centrifuged at 1,000 rpm for 5 min and the cell pellets were resuspended in PBS. Cells were counted with a TC20 automated cell counter (Bio-Rad) and the concentration was adjusted to 1×10^5^ cells/ml in PBS.

### Single-Cell RNA Sequencing

A single-cell state suspension was obtained by pipetting up and down using a glass pipette. Single-cell suspension was then loaded onto a microfluidic chip and scRNA-seq libraries were constructed according to the manufacturer’s instructions (Singleron GEXSCOPE Single Cell RNAseq Library Kit, Singleron Biotechnologies). Sequencing was performed on an Illumina HiSeq X10 instrument with 150-bp paired-end reads.

### Single-Cell RNA Sequencing Alignment and Expression Quantitation

Raw reads were processed to generate gene expression matrices by scopetools (https://anaconda.org/singleronbio/scopetools). Briefly, read 1 contained the cell and molecular barcodes, while all genomic information was contained in read 2. Reads without poly T tails at the intended positions were filtered out, and then for each read, cell barcode and unique molecular identifier (UMI) were extracted. Adapters and poly A tails were trimmed before aligning read 2 to GRCh38 with ensemble version 92 gene annotation. Reads with the same cell barcode, UMI, and gene were grouped together to generate the number of UMIs per gene per cell. Cell number was then determined based on the inflection point of the number of UMI versus sorted cell barcode curve. Finally, the digital gene expression matrix was generated based on the remaining barcode–UMI–gene triplets. In total, we sequenced 28,279 single cells of 7 primary GBM samples.

### Data Filtering, Unsupervised Clustering, Cell-Type Annotation, and Function Analysis

The Seurat package (v.3.2.3) and the DoubletFinder package (v. 2.0.3) in R (v.3.6.3) were applied to filter cells and genes among 28,279 cells. Cells were kept in further data analysis only if they met the following quality control criteria: (i) the number of detected genes was less than twice and more than half the mean number of expression genes across cells coming from the same sample; (ii) expression of mitochondrial genes was less than 20% of total counts in one cell; and (iii) passing the standard workflow of the DoubletFinder package to remove the doublets. Seven samples were merged into one object and clustered without supervision using the harmony package (v.1.0) after filters of cells, and then genes were kept only when they were expressed in at least 10 cells. Uniform Manifold Approximation and Projection (UMAP) was applied to project single cells onto a two-dimensional map to discover heterogeneity among cells. Differentially expressed genes (DEGs) in each cluster were identified by the Seurat function FindMarkers, which can return the gene names, average log fold change, and adjusted *p*-value of genes enriched in every cluster. The package clusterProfiler (3.14.3) was used to accomplish the GO analysis of DEGs, and significant biological processes were picked out by setting “pvalueCutoff=0.05” and “qvalueCutoff=0.05”. Enrichment analysis of specific gene sets was done by the package GSVA (v.1.34.0) by setting “method=ssgsea”.

Malignant tumor cells were distinguished from non-tumor cells by copy number variations (CNVs) as Yuan et al. reported (10). Raw count matrix should first be transformed into log2(counts per thousand molecules +1), and genes that were expressed in less than 100 cells were discarded; subsequently, the average of log2(counts per thousand molecules +1) was computed across the genes on each chromosome; finally, the resulting average of each cell were z-scored and the principal components (PCs) of the resulting z-matrix were calculated. Here, HLA genes on chromosome 6 were also excluded because they could manifest as CNVs in immune cells. For all cells, the first PC yielded the malignant score that can differentiate tumor cells from non-tumor cells. Furthermore, the CNV subclones in different patients were confirmed by the infercnv package (v.1.2.1). Non-tumor cells were annotated to the specific cell types according to the expression of cell marker genes.

### Identification of Tumor Cell Cellular States and Stem-Like Cells

Single-sample gene set enrichment analysis (ssGSEA) (4) was done with the gene signatures for the GBM tumor cell six cellular states (5) and GBM stem-like tumor cell (23) as previously reported compared to a permutated data set (permutation = 1000). The cutoff used here was *p*-value < 0.05. Firstly, tumor cells were annotated as stem-like cell if the *p*-value of stem-like gene set was less than 0.05 and the matching enrichment score was more than 0. Among the remaining un-annotated tumor cells, cancer cells were annotated to the specific cellular state according to the lowest *p*-value when *p*-value was less than 0.05 and related enrichment score was more than 0. If the enrichment score was less than 0, this tumor cell would be marked as un-annotated tumor cells.

### Developmental Linage of Six Cellular States

The velocyto python package was applied to recount the spliced reads and unspliced reads based on previously aligned bam files, then the velocyto.R package (v.0.6) was used to calculate RNA velocity values for each gene from each cell and embed RNA velocity vector to the 2-D diffusion map space.

### Construction of Regulon Network

Simultaneous gene regulatory networks of the six tumor cell cellular states and non-tumor cells were constructed by the SCENIC package (v.1.1.1.10 and v.1.2.2). The databases used were “hg19-500bp-upstream-7species.mc9nr.feather” and “hg19-tss-centered-10kb-7species.mc9nr.feather”. Genes were included in analysis only if they were expressed in at least 10 cells and were contained in the former two databases. The regulon specificity score was calculated by the function calcRSS.

### Analysis of Public GBM Datasets

The mRNA expression data and metadata containing survival information for TCGA and Chinese Glioma Genome Atlas (CGGA) GBM patients were downloaded from http://www.cbioportal.org/ and http://www.cgga.org.cn/, respectively. We ranked the GBM patients from high to low according to their enrichment scores of specific cell-type marker signatures, then labeled the upper 50% of the patients as the higher group and the lower 50% of the patients as the lower group. Survival curves were performed by Kaplan–Meier analysis in the package survival (3.2-7) between the higher and the lower group, and were tested for significance using the Mantel-Cox log-rank test. A value of *p* < 0.05 was considered statistically significant.

### Cell-to-Cell Interactions

Cross-talks between the GBM tumor cell six cellular states and other microenvironmental cells were done using the CellChat package (v.0.5.5), and CellChatDB.huma was used as the ligand–receptor interaction reference database. The function computeCommunProbPathway inferred the cell–cell communication at a signaling pathway level, and then we explored how signaling pathways coordinate together among multiple cell types by using the function identifyCommunicationPatterns.

### Investigating the Role of Six Cellular States in TAM M2-Type Polarization

We did NicheNet (v.1.0.0) analysis to link ligands secreted by the six tumor cell cellular states to TAM M2-type marker genes (**Supplementary Figure 8C**). The ligand–target prior model, database for ligand–receptor network, and weighted integrated network were provided by NicheNet. If the gene was detected in at least 10% of cells among the same cellular state or TAMs, it was considered as expressed gene and was used in this part analysis. We computed the ligand activity compared to the background set of genes and ranked ligands based on the presence of their target genes in the M2-type marker gene sets. In the ligand–receptor network analysis, only bona fide ligand–receptor interactions documented in literature and publicly available databases were remained.

### Immunofluorescence

Formalin-fixed paraffin-embedded sections of primary GBM were collected from the same 7 patients whose samples underwent scRNA-seq in this study. The protein expression levels of the marker genes were detected by immunofluorescence for human primary GBM specimens with antibodies shown in **Supplementary Table 7**. The samples were incubated with the first primary antibody against *CD14* (1:200 for IF, Servicebio) overnight at 4°C and then with the first corresponding secondary antibody at room temperature for 50 min under dark conditions. Later, the sample slides were incubated with the second antibody *ERO1A* (1:200 for IF, DF12984) overnight at 4°C and then with the second corresponding secondary antibody at room temperature for 50 min under dark conditions. Slides were counterstained with DAPI for nuclei visualization. Finally, the slides were imaged using Imaging System from Nikon. We used CaseViewer software (3DHISTECH) to unmix and remove auto-fluorescence and to analyze the multispectral images.

## Results

### Dissecting GBM Cellular States and Correlation With TCGA Subtypes

**Figure 1:**
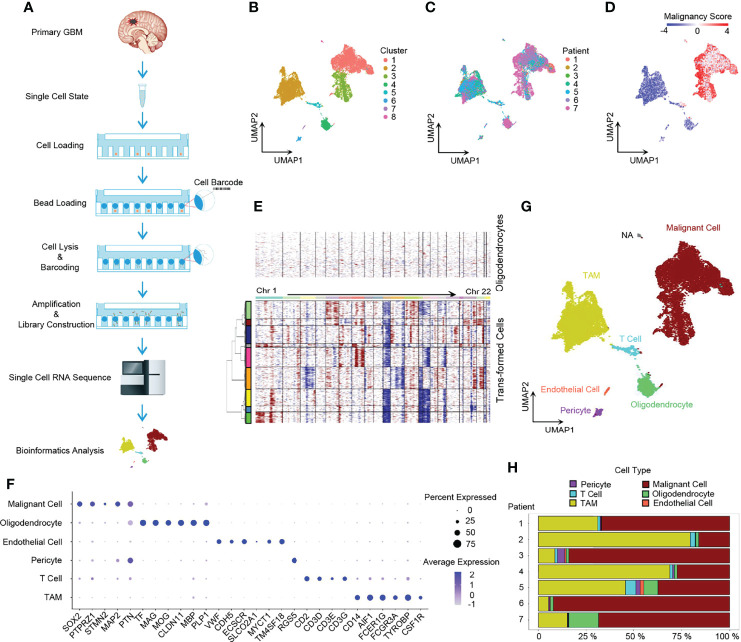
Dissection of primary GBM by scRNA-seq. **(A)** Scheme of the workflow in our study. **(B)** UMAP projection of all 20,289 GBM cells including tumor cells and stromal cells. Eight clusters were found when index Resolution in Seurat FindClusters was set as 0.05. **(C)** UMAP visualization showing cells of individuals. **(D)** UMAP of malignancy scores. Transformed cells had higher malignancy scores and were colored with red, while non-tumor cells had lower malignancy scores and were in blue. **(E)** Evaluation of copy number variations (CNVs). Compared to oligodendrocytes, malignant cells presented with obvious CNVs. Red, amplification; blue, deletion. **(F)** Dot plot of cell marker gene expression level. **(G)** Annotation of cell types in GBM. The majority of GBM cells were malignant cells and TAMs, and GBM still had a small number of normal oligodendrocytes, endothelial cells, pericytes, and T cells. **(H)** Composition ratio of cell types in individual GBM. See also **Supplementary Figure 1**.

We conducted single-cell 3’ mRNA sequencing of 7 GBM patient samples and obtained 28,279 single-cell transcriptomes at a depth of 100,000 mean reads per cell (**Figure 1A** and **Supplementary Tables 1**, **2**). The median number of genes detected per sample ranged from 1,053 to 2,098. In total, 25,467 genes in 20,289 cells passed the quality control filtering (see the *Methods* section) and were used in further downstream data analysis. The whole transcriptome of all single cells after batch effect correction was used to perform unsupervised clustering analysis and the results were visualized using the UMAP for dimension reduction (**Figure 1B**). The distribution of single cells from different patients was also shown (**Figure 1C**).

Firstly, we dissected the GBM cell composition. Large CNVs and aneuploidies are readily detected by scRNA-seq and can be applied to distinguish malignantly transformed tumor cells from non-malignant cells (22). We adopted a computational pipeline reported previously (10) to calculate the malignancy score based on CNVs, which was subsequently used to identify GBM tumor cells. When compared to normal oligodendrocytes, malignant cells had higher malignancy scores (**Figure 1D**) with distinct CNVs (**Figure 1E**). Furthermore, non-tumor cells were annotated to specific cell types by marker genes (**Figures 1F, G**) (5, 24). Notably, *SOX2* was pervasively expressed in tumor cells (**Figure 1F** and **Supplementary Figure 1B**), which is consistent with the previous study (10). Compared to non-malignant oligodendrocytes, tumor cells usually exhibited a loss of chromosome 10 (**Figure 1E**), which is the earliest and one of the most common genetic alterations in adult GBMs (25). All GBM patients had their own main unique CNV subclones, indicating the existence of genetically heterogeneous malignant cells (**Supplementary Figure 1A**). The majority of cells were malignant tumor cells and TAMs (**Figure 1H**).

**Figure 2:**
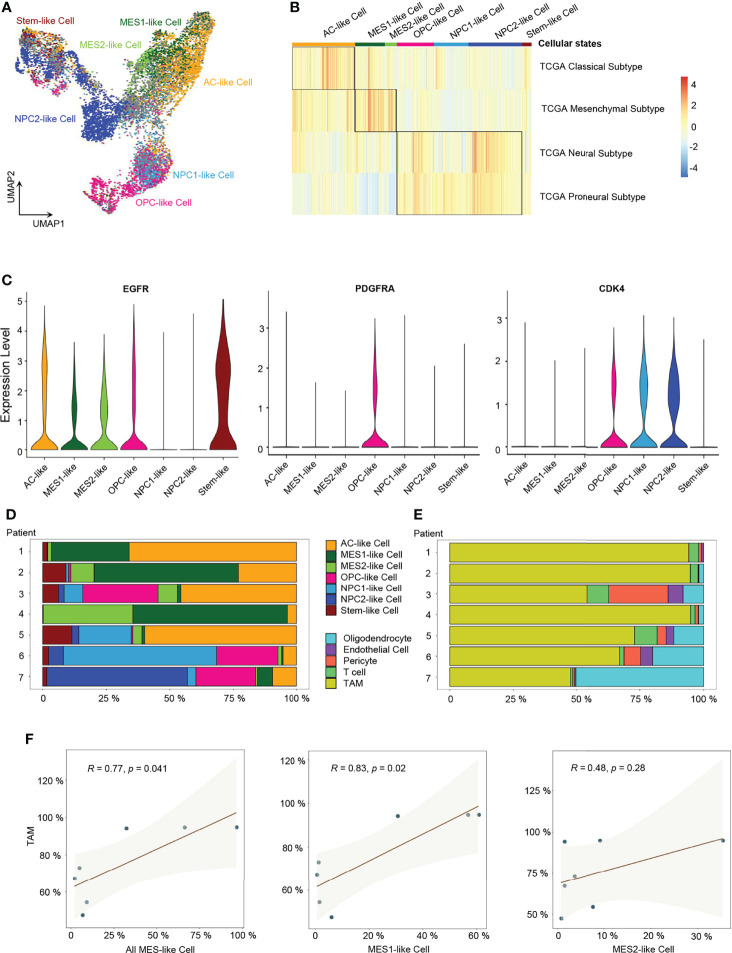
Identification of six GBM tumor cell cellular states. **(A)** UMAP projection of the six GBM tumor cell cellular states and stem-like cells. **(B)** Heatmap of the four GBM TCGA transcription subtype scores. **(C)** Violin plot of *EGFR*, *PDGFRA*, and *CDK4* expression level in different cellular states. **(D)** Composition ratio of the six cellular states in tumor cells. **(E)** Composition ratio of stromal cells in non-tumor cells. **(F)** Correlation between the number of MES-like cells and TAMs. See also **Supplementary Figure 2** and **Supplementary Table 3**.

Next, we performed ssGSEA with the gene meta-modules (5) and identified the six cellular states, namely, AC-like, MES1-like, MES2-like, OPC-like, NPC1-like, and NPC2-like states. We found that nearly 72% of malignant cells can be successfully annotated to one of the six specific cellular states with *p*-value < 0.05 (**Figure 2A** and **Supplementary Figure 2B**; **Supplementary Table 3**), whereas 28% of cells had gene signatures associated with multiple cellular states, suggesting the existence of a developmental lineage continuum within the tumor cell compartment. To correlate the transcriptional cellular states to TCGA GBM subtypes defined by genomic alterations, we also performed the ssGSEA analysis with the gene signatures of the TCGA GBM genomic subtype. Our results revealed that AC-like cells were correlated to TCGA-classical subtype (**Figure 2B**) with higher expression of *EGFR* (**Figure 2C**). MES-like cells were enriched for the TCGA-mesenchymal subtype (**Figure 2B**). We observed that the tumors in patients 1, 2, and 4 with a higher percentage of MES-like cells also contained a higher proportion of TAMs (**Figures 2D, E**). Thus, as shown in the TCGA-mesenchymal subtype data, MES-like cells were correlated with infiltrating TAMs (**Figure 2F**). However, no significant difference was observed between OPC-like and NPC-like states in the enrichment score between the TCGA-neural subtype and TCGA-proneural subtype (**Figure 2B**), indicating an overlap of TCGA-proneural and TCGA-neural subtypes at the transcriptional level.

### Developmental Trajectory, Lineage Analysis, and Cells of GBM Origin

**Figure 3:**
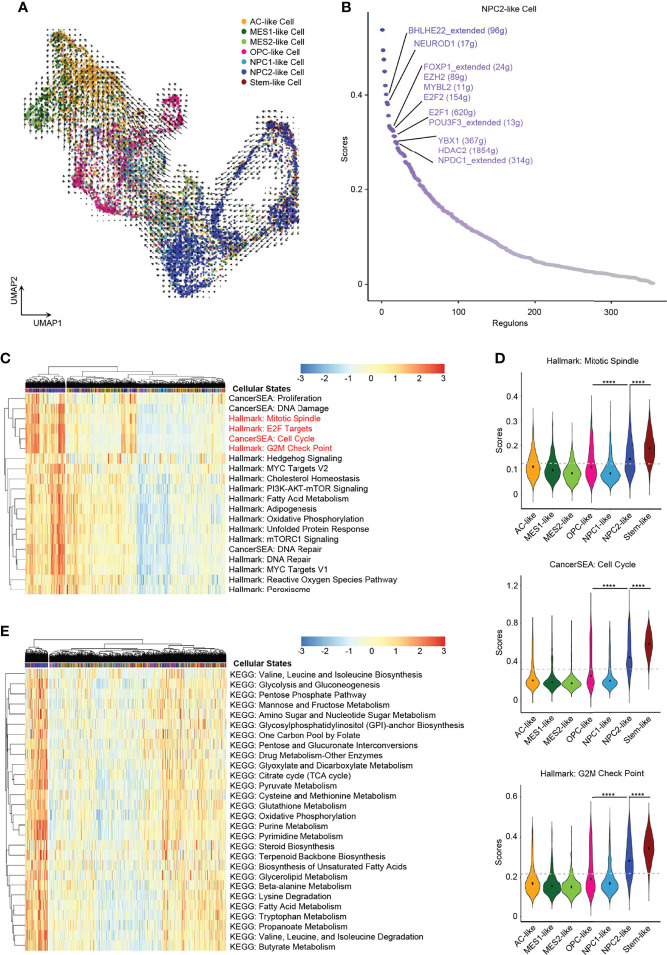
NPC2-like cells, the original root cell of GBM. **(A)** Inferred developmental trajectory of the six GBM tumor cell cellular states by RNA velocity, which implied that the NPC2-like cells, like stem-like cells, were the root cell of the developmental trajectory. **(B)** The activated regulons ranked by regulon specificity score from high to low in the NPC2-like cells. **(C)** Heatmap of cancer-specific gene sets in the six cellular states and stem-like cells. **(D)** Violin plot of the Mitotic Spindle, Cell Cycle, and G2M Check Point gene set scores in the six cellular states and stem-like cells. NPC2-like cells, like stem-like cells, were in the cell cycle. **(E)** Heatmap of metabolism gene sets in the six cellular states and stem-like cells. NPC2-like cells had a higher metabolism level than other cellular states. Gray dash line, average score; ****p < 0.0001. See also **Supplementary Figure 3** and **Supplementary Table 4**.

In order to explore the developmental lineages of the six cellular states of GBM tumor cells, we used RNA Velocity to construct the trajectory (26). All tumor cells from 7 patients were integrated together to construct the developmental trajectory because not all patients contained all the six cellular states (**Figure 2D**). Apart from the six cellular states, GBM stem cells were annotated individually (**Figure 2A**), which can also help us to confirm the root cell of origin. In the RNA Velocity lineages, we found that NPC2-like cells and GBM stem-like cells were at the root of the developmental tree, which implied that NPC2-like tumor cells could be the cells of origin among all six cellular states in GBM (**Figure 3A** and **Supplementary Figure 3**). Next, we analyzed the transcription factor (TF)-mediated gene regulatory networks using regulon, a gene set that is regulated as a unit, in the NPC2-like cells (**Figure 3B**). Some of these top activated TFs were related to cell cycle (e.g., *E2F1*, *E2F2*, *MYBL2*, and *YBX1*) (27); cell fate determination, proliferation, and differentiation (e.g., *BHLHE22*, *HDAC2*, *NEUROD1*, and *NPDC1*) (28); nervous system development (*POU3F3*); and proneural-stem marker (*EZH2*) (29). This implied that NPC2-like tumor cells were in the proliferative state and could be the cells of origin in the tumor cell lineages. Then, we further conducted enrichment analysis of cancer-related gene sets, and these results were consistent with the former finding that NPC2-like GBM cells were in cell cycle (**Figures 3C, D**). Every cellular activity requires energy, and if one cell is in proliferation and cell cycle, it needs more energy than the quiescent cell. Thus, we compared the metabolic level among the six cellular states and found that the NPC2-like cells had higher metabolic activities as compared to other tumor cell states, for example, with elevated citrate cycle (TCA cycle), oxidative phosphorylation, and fatty acid metabolism (**Figure 3E**). Thus, the NPC2-like cells in proliferative state with high metabolic activity could be the cells of origin in the developmental trajectory of human GBM.

### Subtype-Specific Immune Mediators and Hypoxia-Response Tumor Cells

**Figure 4:**
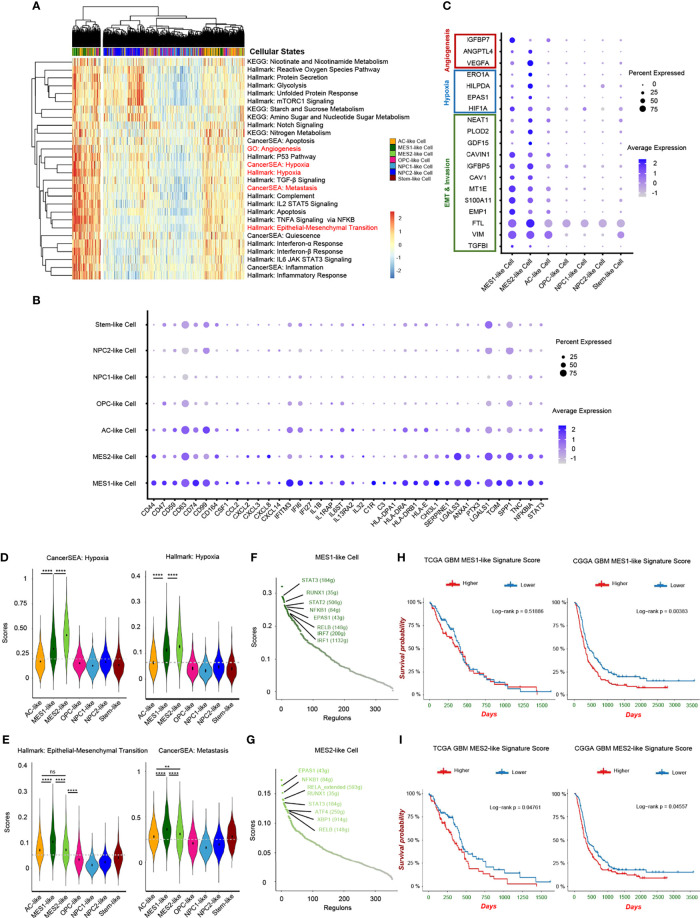
Hypoxia-response tumor cells in GBM. **(A)** Heatmap of cancer-specific gene sets in the six cellular states and stem-like cells. **(B)** Upregulated immune gene pattern in MES1-like and MES2-like cells. **(C)** High expressed genes related to angiogenesis, hypoxia, and invasion in MES1-like and MES2-like cells. Violin plot of the hypoxia **(D)**, and epithelial–mesenchymal transition and metastasis **(E)** gene set scores in the six cellular states and stem-like cells. MES1-like and MES2-like cells were the hypoxia-response tumor cells in GBM. The activated regulons in the MES1-like **(F)** and MES2-like** (G)** cells ranked by regulon specificity score from high to low. Survival analysis of the MES1-like **(H)** and MES2-like **(I)** cell signatures in TCGA and CGGA GBM databases, respectively. Gray dash line, average score; ns, no significance; **p < 0.01; ****p < 0.0001. See also **Supplementary Figure 4** and **Supplementary Table 4**.

Although recent single-cell studies determined that GBMs consist of diverse cellular states, we still do not know how different tumor cellular states differentially affect the TME and the potential impact on the prognosis of GBM patients. Herein, we conducted a cancer subtype-specific gene set enrichment analysis and found that MES1-like and MES2-like tumor cells were associated with the hypoxia niche (**Figures 4A, D**). Another characteristic of MES-like cells was the induction of immune mediators, including activation of *IL2/STAT5* Signaling, *TNFA* Signaling *via NFKB*, *IL6/JAK/STAT3* Signaling, Interferon-α Response, and Interferon-β Response. Furthermore, MES1-like and MES2-like tumor cells had high expression levels of immune factors (**Figure 4B**) (e.g., *CSF1*, *CCL2*, *CXCL2*, *CXCL3*, *CXCL8*, *CXCL14*, *IFITM3*, *IFI6*, *IFI27*, *IL1B*, *IL1RAP*, *IL6ST*, *IL13RA2*, and *IL32*), which play important roles in the formation of an immunosuppressive TME (21). This was consistent with the former result that the MES-like cells were correlated with infiltrating TAMs, which can promote the immunosuppressive environment and tumor progression. Although the MES-like cells were in quiescence state, non-cycling, they were associated with an invasion-promoting microenvironment with elevated *TGF-β* signaling activation and epithelial–mesenchymal transition (EMT) (**Figures 4A, C, E**). These results suggested that MES1-like and MES2-like cellular state cells were the GBM tumor cells that produce soluble mediators to modulate the TME and potentially lead to poor prognosis.

Then, we constructed the regulon networks in MES1-like and MES2-like state cells. Although both *HIF1A* and *EPAS1* genes were upregulated in MES1-like and MES2-like tumor cells (**Figure 4C**), only *EPAS1* regulon (not *HIF1A* regulon) was activated in both MES1-like and MES2-like cells among the top activated regulons (**Figures 4F, G**). While cells respond to chronic hypoxia *via* the *EPAS1* pathway, the *HIF1A* pathway is activated when an acute decrease of oxygen level (30). These results suggested that MES-like tumor cells were in a chronic hypoxia environment. *STAT3* is one of the major mediators of tumor-induced immunosuppression and was activated in MES-like state tumor cells, and *NFKB1*, an inflammatory regulon, was also upregulated in MES-like cells. Therefore, these cells were likely related to an immunosuppressive microenvironment. Other top activated regulons, such as *RELB* and *RUNX1*, are oncogenic drivers of mesenchymal GBM subtype and contributed to EMT *via* the *TGF-β* pathway (31, 32). Results of enrichment analysis and regulon networks coincided in MES1-like and MES2-like cells, suggesting that it was MES-like cellular state tumor cells that gave rise to the TME known to be associated with poor clinical outcomes. This was confirmed by using public GBM datasets, TCGA and the CGGA (**Figures 4H, I**). GBM patients with a lower MES-like signature score had longer survival time than those with a higher score.

### Heterogeneity of TAMs in GBM

**Figure 5:**
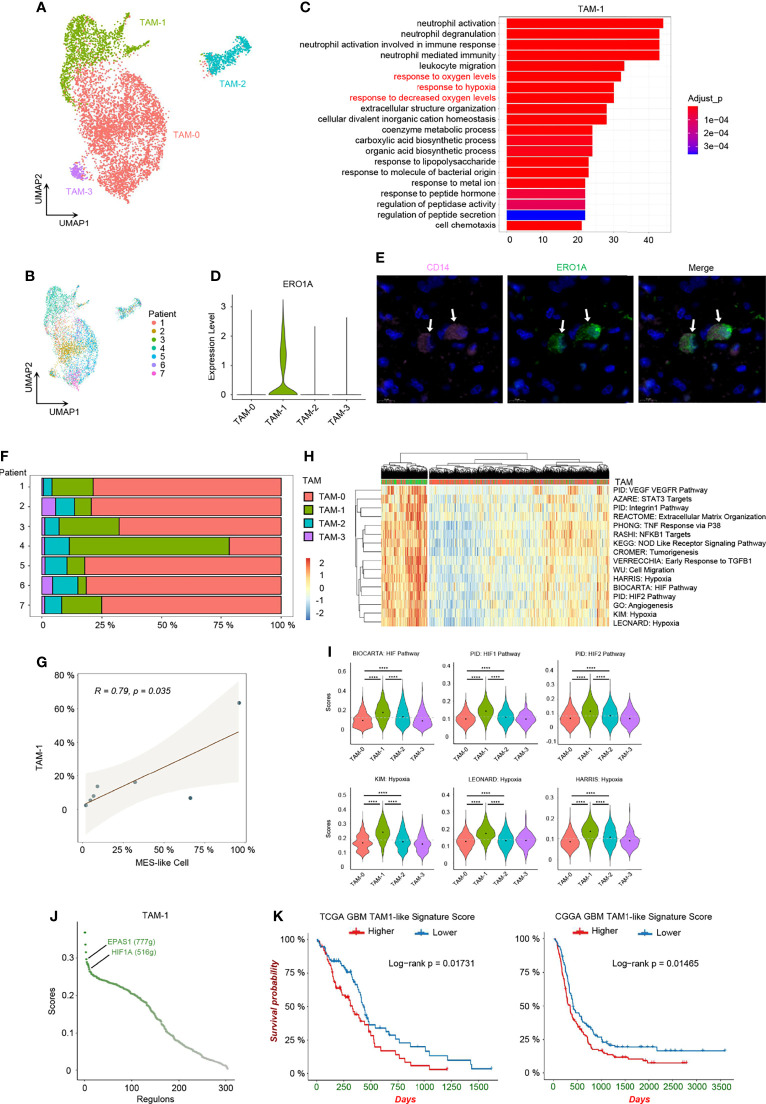
Hypoxia-response TAMs in GBM. **(A)** UMAP visualization of TAMs. **(B)** UMAP visualization showing TAMs of individuals. **(C)** Top 20 function analysis results of TAM-1 cluster. Hypoxia-related biological processes were colored in red. **(D)** Violin plot of *ERO1A* expression level in different TAM clusters. **(E)** Immunofluorescence staining for TAM-1 cluster (*CD14+ERO1A+*) in patient tumor sample. The staining was performed for seven patients, one section each, and a representative image from patient 6 with TAM-1 pointed out by white arrows was shown; scale, 10 μm. The other images are shown in Supplementary **Supplementary Figure 6**. **(F)** Composition ratio of the TAM clusters in individual GBM. **(G)** Correlation between the number of MES-like and TAM-1 cells. **(H)** Heatmap of specific gene sets in TAMs. **(I)** Violin plot of the hypoxia gene set scores in TAMs. TAM-1 was the hypoxia-response cluster in TAMs. **(J)** The activated regulons in the TAM-1 cluster ranked by the regulon specificity score from high to low. **(K)** Survival analysis of the TAM-1 cluster signatures in TCGA and CGGA GBM databases, respectively. Gray dash line, average score; ****p < 0.0001. See also **Supplementary Figures 5** and **6**, and **Supplementary Tables 3** and **6**.

While infiltrating macrophages and activated microglia are the primary immune cells that reside in and around the glioma TME, there is no clear distinction between them and it is still difficult to distinguish these two cell types due to their common myeloid lineage origin (10, 33). We noticed that TAMs expressed not only macrophage genes, but also microglia markers, and TAMs distributed together in the principal component analysis (PCA) reduction analysis based on the macrophage and microglia markers (**Supplementary Figures 5A–C**). Additionally, a significant correlation in the enrichment scores between macrophage and microglia marker genes in TAMs was uncovered (**Supplementary Figure 5D**). Therefore, we used TAMs, namely, tumor-associated macrophage/microglia, in our study, as these two immune cells are difficult to distinguish and are functionally similar in GBM. TAMs made up the majority of GBM stromal cells. We discovered an inconsistent expression pattern in malignant cells as well as TAMs from the heatmap of top DEGs (**Supplementary Figure 1B**), indicating that TAMs were also heterogeneous in nature and depended on GBM subtypes. Herein, we identified 4 TAM clusters with different expression patterns using clustering and reduction (**Figure 5A**) and TAMs were also shown from different individuals (**Figure 5B**). These TAM clusters had distinct transcriptional profiles and associated functions. TAM-0 cluster was related to cytokine production and lipoprotein metabolism with high expression levels of chemokines (e.g., *CCL3*, *CCL4*, *CCL3L1*, and *CCL4L2*) and lipoprotein receptors (e.g., *APOE*, *APOC1*, and *OLR1*) (**Supplementary Figures 5E, F**). *MKI67+* TAMs, namely, the TAM-2 cluster, were in cell cycle, and overexpressed other cell cycle-related genes, such as *TOP2A*, *CENPF*, and *NUSAP1* (**Supplementary Figures 5E, F**). TAM-3 cluster had high expression levels of *RSAD2*, *IFIT1*, *IFIT2*, *IFIT3*, and *ISG15*, and could respond to interferon in GBM (**Supplementary Figures 5E, F**). The TAM-1 cluster, which responded to decreased oxygen levels in GBM, was also identified (**Figures 5C, H, I**), which is of particular interest. After revealing the heterogeneity in TAMs, we set out to investigate which types of these TAMs could affect the TME and potentially the survival of patients.

### Hypoxia Niches and TAM-1 Signature in Prognosis

Firstly, we confirmed the existence of the TAM-1 cluster by scRNA-seq in mRNA level and using immunohistochemistry to verify the protein marker expression (*CD14+ERO1A+*) (**Figures 5D, E** and **Supplementary Figures 6**). We further compared the relationship between MES-like cellular state and different TAM clusters, because the quantity of MES-like tumor cells and all TAMs were positively correlated (**Figure 2F**). In particular, there was a significant association between MES-like cells and the TAM-1 cluster (**Figures 5F, G** and **Supplementary Figure 5G**), and they were both related to the GBM hypoxia niche. Thus, we speculated that the hypoxia-response TAMs, namely, the TAM-1 cluster, could associate with poor prognosis. By constructing the regulon networks in TAMs, we found that hypoxia-related regulons, *EPAS1* and *HIF1A*, were both activated in the TAM-1 cluster (**Figure 5J** and **Supplementary Table 5**), which differs from the hypoxia response in MES1-like and MES2-like tumor cells. This suggested that the GBM hypoxia niche could be divided into two conditions, namely, acute and chronic hypoxia microenvironments: MES-like tumor cells were only in the chronic hypoxia niche, while TAM-1 distributed in both hypoxia niches. However, the TAM-1 cluster signature was also enriched in the process of invasion and extracellular matrix organization (**Figure 5H** and **Supplementary Figure 5H**). Thus, TAM-1 was involved in the hypoxia and progressively invasive niche as well. Ultimately, we checked the differential survival curves of patients in relation to different TAM clusters in the TCGA and CGGA GBM database (**Figure 5K** and **Supplementary Figures 5I–K**). We observed that it was the TAM-1 cluster that is mostly significantly associated with poor prognosis. Patients with a lower TAM-1 signature score had a longer survival time than those with a higher score.

### Hypoxia-Specific Inter-Cellular Communication and Angiogenesis

**Figure 6:**
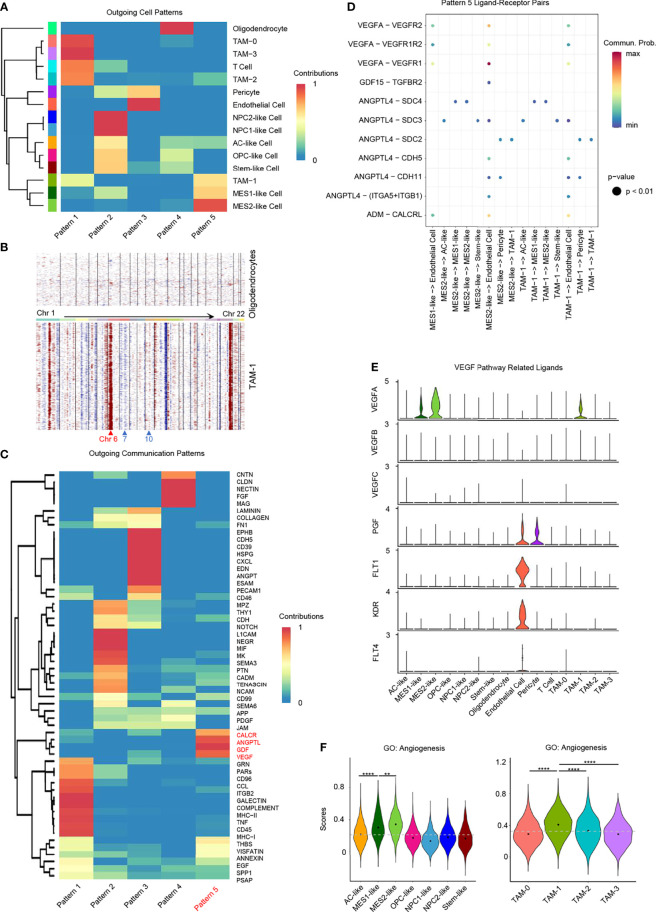
Role of hypoxia-response cells in angiogenesis. **(A)** Cluster of GBM cell types according to the source cell functions in cell–cell communication. Hypoxia-response cell types, namely, MES1-like, MES2-like, and TAM-1 cells, were in the same pattern 5. **(B)** Compared to oligodendrocytes, TAM-1 cells presented with obvious CNVs in chromosome 6, but no CNVs in chromosomes 7 and 10. Red, amplification; blue, deletion. **(C)** Cell–cell communication-related pathways in different outgoing cell patterns. **(D)** Dot plot of outgoing cell pattern 5-related ligand–receptor pairs. **(E)** Violin plot of *VEGF* pathway-related ligand expression levels. **(F)** Violin plot of the angiogenesis gene set scores in GBM tumor cells and TAMs, respectively. Hypoxia-response cells, namely, MES1-like, MES2-like, and TAM-1 cells, had the highest score. Commun., communication; Prob., probability; gray dash line, average score; **p < 0.01; ****p < 0.0001. See also **Supplementary Figure 7**.

We identified a hypoxia-specific intercellular communication network and the potential impact on promoting angiogenesis. Firstly, we identified significant ligand–receptor interactions between different cell types using CellChat (34). Then, we inferred cell–cell communication at a signaling pathway level from ligand–receptor pairs (**Supplementary Figures 7A, B**). Finally, these cell–cell communication signaling pathways were clustered to generate the cell–cell specific communication pattern (**Figure 6A** and **Supplementary Figure 7C**).

Pattern 5 was revealed to be specific to these GBM hypoxia-response cells, namely, MES1-like tumor cells, MES2-like tumor cells, and TAM-1; the remaining immune cells were grouped in pattern 1, and the remaining tumor cellular states were clustered together in pattern 2 (**Figure 6A**). As the communication pattern from endothelial cells to other cell types was similar to pericytes, they were in the same group, pattern 3 (**Figure 6A**). Oligodendrocyte was the only normal glial-lineage cell type; thus, it was different from other GBM cell types in cellular communication pattern (**Figure 6A**). Because TAM-1 and MES-like cells were clustered in the same pattern, we further checked the purity of TAM-1 to rule out the possibility that TAM-1 was formed as doublets of tumor cells and TAMs even though the standard pipeline of DoubletFinder was taken into the data filtering process (35). Apart from the malignancy score (**Figure 1D**), we also constructed the CNVs in TAM-1 compared to oligodendrocytes without filtration of HLA genes. TAM-1 cells had amplification in chromosome 6, which reflected the TAMs with high expression of HLA genes, but no CNVs in chromosome 7 and 10 which was different from tumor cells (**Figures 1E**, **6B**). From these results, we confirmed the purity of the TAM-1 cluster. Next, we wanted to clarify the former finding from the cellular interaction perspective that GBM hypoxia-response cells contributed to the TME associated with poor survival of GBM patients. Pattern 5 included *CALCR*, *ANGPTL*, *GDF*, and *VEGF* pathways (**Figure 6C**). We further deciphered the significant ligand–receptor pairs in pattern 5, and the majority of these interactions were from source cells targeting endothelial cells (**Figure 6D**). In addition, we found that these ligands secreted by hypoxia-related GBM cell types, namely, MES1-like tumor cells, MES2-like tumor cells, and TAM-1, may stimulate angiogenesis *via*, for example, *ADM*, *ANGPTL4*, *GDF15*, and *VEGFA* (36–38). Then, we also explored the expression of *VEGF* pathway-related ligands, because *VEGFA* is the principal agonist during the formation of vasculature. We discovered that only MES1-like tumor cells, MES2-like tumor cells, and TAM-1 expressed the *VEGFA* in GBM (**Figure 6E**). These results indicated that hypoxia-dependent GBM cell types promoted angiogenesis (**Figures 4A**, **5H**, **6F**), because solid tumors are unable to grow beyond a couple of millimeters without neo-vascularization providing oxygen and nutrients to tumor cells. Extensive tumor angiogenesis and endothelial proliferation is a hallmark of GBM, and tumor vascularity is significantly correlated with poor survival (39). In short, hypoxia-specific cellular communication attributed in part to these hypoxia-response GBM cell types could induce poor outcomes in GBM patients.

### Role of GBM Tumor Cells in TAM M2-Type Polarization

**Figure 7:**
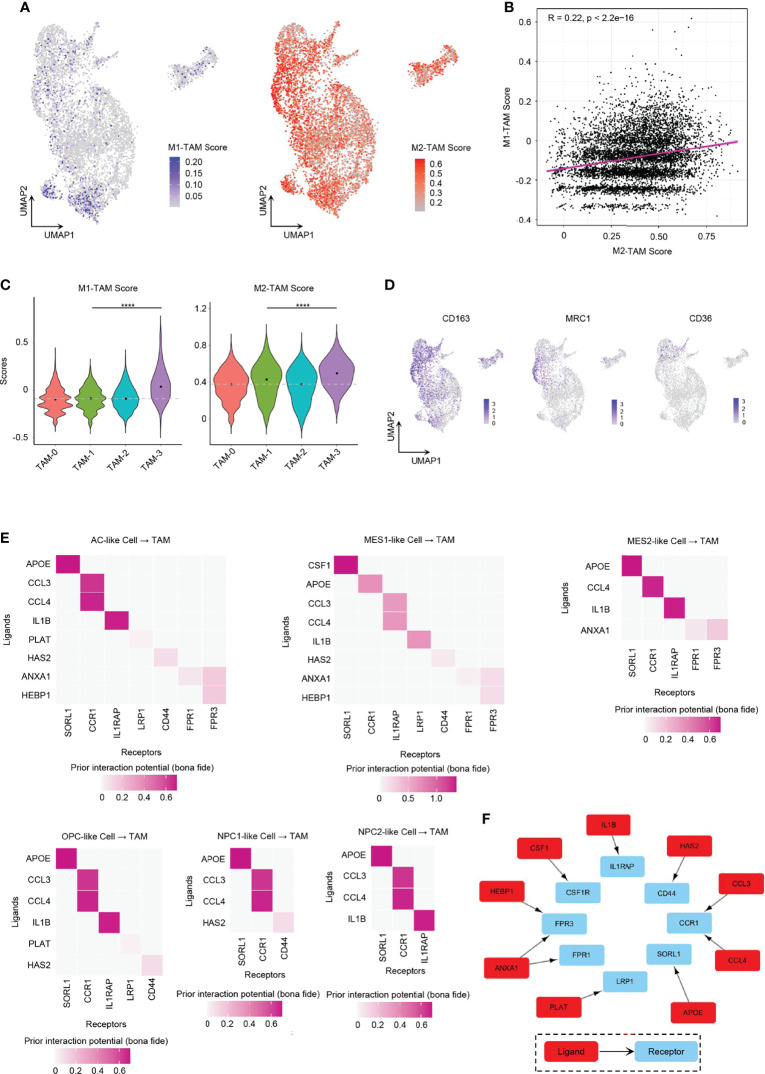
Role of GBM tumor cells in promoting TAM M2-type polarization. **(A)** UMAP projection showed the M1-TAM score and M2-TAM score in TAMs. **(B)** Correlation between M1-TAM score and M2-TAM score in TAMs. **(C)** Violin plot of the M1-TAM score and M2-TAM score in different TAM clusters. **(D)** Expression levels of M2-TAM marker genes, namely, *CD163*, *MRC1*, and *CD36*, in TAMs. **(E)** Ligand–receptor pairs from different GBM tumor cellular state cells to TAMs took part in TAM M2-type polarization. **(F)** Ten L–R pairs, in total, could promote TAM M2-type polarization. Gray dash line, average score; ****p < 0.0001. See also **Supplementary Figure 8**.

We found it hard to divide TAMs into the M1 or M2 phenotype. While TAMs had relatively higher enrichment score of M2-type TAM marker genes than M1-type TAM marker genes (**Figures 7A–D**), GBM TAMs still over-expressed some markers of M1-type TAMs, such as *TSPO*, *CD86*, and *IL1B* (**Supplementary Figure 8A**), which was consistent with literature (17). Considering that TAMs had mixed M1/M2 phenotypes, we took a method that predicts the ligand–target links from GBM tumor cells to TAMs based on scRNA-seq data (40). The expression of each TAM M2-type marker gene used in the analysis is listed in **Figure 7D** and **Supplementary Figure 8B**. We discovered that tumor cells from all six GBM cellular states secreted ligands, which may target TAM M2-type marker genes to induce activation (**Supplementary Figure 8C**). Thus, these TAMs would gradually shift to an M2-like phenotype and then may promote GBM progression. It was found that MES-like tumor cells had a higher number of promoting ligands than other cellular states (**Supplementary Figure 8C**), which also explained in part why MES-like tumor cells correlate with poor prognosis (**Figures 4H, I**). To clarify the cross-talk between different cellular states and TAMs, we linked the ligands secreted by the six cellular states of GBM and receptors of TAMs (**Figure 7E**). Because many ligand–receptor (L–R) pairs were speculated, we only chose the L–R networks that have been reported in literature and publicly available databases. We observed that ten L–R pairs may take part in the M2-type polarization of TAMs (**Figure 7F**).

One of these L–R pairs has been confirmed experimentally in human glioma. *CSF1* was reported for recruitment and polarization of TAMs in several cancers, and receptor inhibition of *CSF1* in GBM could block TAMs from M2-type polarization and inhibit tumor progression (12, 41). We found that only MES1-like tumor cells secreted *CSF1* that interacted with *CSF1R* on TAMs.

Some of the L–R axes have also been reported in other cancers. *ANXA1* is an immune-modulating protein that plays a central role in the anti-inflammatory and neuroprotection in brain (42). The *ANXA1–FPR2* axis between tumor cells and TAMs may enhance cancer cell growth and migration by promoting M2-type polarization of TAMs, and furthermore, the *ANXA1*-deficient breast cancer mouse model showed enhanced survival due to increased M1 TAMs within the tumor environment (43). However, *ANXA1–FPR1* and *ANXA1–FPR3* pairs (not *ANXA1–FPR2* pairs) were found to be involved in the polarization process in our results. AC-like and MES-like tumor cells expressed ligand *ANXA1*. Tumor cell-derived *IL1B* cross-talks with *IL1RAP* in TAMs could establish an immunosuppressive environment by activating M2 TAMs in pancreatic cancer, which required *NF-κB* activation (16). All tumor cells except NPC1-like cells expressed the ligand *IL1B*. Various tumor cell types produce *CCL4* that has been shown to promote colon cancer progression through inducing M2 TAM infiltration together with other chemokines such as *CCL3* (44). As *CCR1* exhibits nearly 100-fold lower affinity for *CCL4* than for *CCL3*, *CCR5* is the specific receptor for *CCL4* (45). However, in our analysis, it showed that both *CCL3* and *CLL4* interacted with *CCR1* (not *CCR5*) in human GBM. All six GBM cellular states expressed *CCL3* and *CCL4*. Tumor cell-associated hyaluronan (HA) and the associated extracellular matrix trigger TAM M2-like polarization *via* CD44 in breast cancer (46).

The remaining L–R signal pathways are documented in other diseases or have not been reported in M2 polarization. *APOE* can downregulate M1 phenotype macrophage markers and upregulate markers of anti-inflammatory M2 macrophages *via* surface *APOE* receptors in the development of atherosclerosis (47). Further experimental works still need to be done to confirm the role of *PLAT* and *HEBP1* in TAM polarization.

## Discussion

Genetic, epigenetic, and microenvironmental cues drive GBM heterogeneity, which remains one of the greatest barriers for therapy. Previous work uncovered that GBM tumor cells could be mapped to six dominant cellular states (AC-like, MES1-like, MES2-like, OPC-like, NPC1-like, and NPC2-like) with specific gene expression signatures (5). Our work further explored the correlation of six transcriptional cellular states to GBM TCGA subtypes, developmental lineages, regulon networks, and cell–cell communication with stromal cells, which can link single-cell transcriptional states to GBM genotypes, improving our understanding of intratumor heterogeneity and the differential roles of tumor subtypes in shaping TME. Although previous studies have focused on malignant cells in GBM (8, 9), it is believed that stromal cells including immune and vascular cells also play essential roles in tumor development and progression. All these cell types were included in our study, allowing us to explore cell-to-cell communications between GBM tumor cells and stromal cells in a subtype-specific manner. Our results provided the first systematic portrait at the single-cell level of the differential roles of six GBM cellular states, dissected the heterogeneity of TAM, and revealed the unique mechanisms in driving M2-type polarization of TAMs.

In 2010, TCGA classified GBM into four genotypes based on genetic alterations, but the transcriptomic profiles from each subtype were also obtained by bulk RNA sequencing (3). Xiao assigned human GBM scRNA-seq data to four TCGA GBM subtypes, but only 33% of tumor cells were annotated (48). In our study, we were able to identify 72% of GBM tumor cells that could be successfully annotated to unique GBM cellular states. The improvement of subtype-specific annotation may be affected by technical issues such as high dropout rates in scRNA-seq as well as the intrinsic heterogeneity within the continuum of tumor cell lineage differentiation trajectories. As reported, functional gene set enrichment analysis of MES-like cells is related to VERHAAK_GLIOBLASTOMA_MESENCHYMAL; enrichment analysis of OPC-like cells and NPC-like cells is related to VERHAAK_GLIOBLASTOMA_PRONEURAL (5). Our results also confirmed that MES1-like and MES2-like cells correlated with the TCGA-mesenchymal subtype, and OPC-like, NPC1-like, and NPC2-like cells were related to TCGA-proneural subtypes. Furthermore, we found that AC-like tumor cells were similar to the TCGA-classical subtype and expressed high levels of *EGFR*. Recently, researchers suggested the removal of the TCGA-neural subtype due to its overlap with the TCGA-proneural subtype (4, 23), and this overlap was also reflected in the overlapping relationship with OPC-like, NPC1-like, and NPC2-like cells in our study. We observed that individual GBM samples contained at least three cellular states and their own unique CNV subclone groups, suggesting a high degree of intratumor and inter-tumor heterogeneity of GBM.

Previous studies on the origin of glioma cells indicated that neural progenitor cells, oligodendrocyte progenitor cells, and astrocytes, upon pathological insult, all have the ability to induce tumorigenesis (49); Neftel and colleagues demonstrated GBM cellular transition by comparing the cellular composition of the GBM mouse PDX model (5). Through developmental trajectory analysis using RNA velocity in our study, NPC2-like state cells developed into other tumor cellular states. Many upregulated regulons in NPC2-like cells are correlated with cell cycle and proliferation (e.g., *E2F1*, *E2F2*, *MYBL2*, *YBX1*, *BHLHE22*, *HDAC2*, *NEUROD1*, *NPDC1*, and *POU3F3*). Because tumor cell proliferation and invasion are stochastically mutually exclusive events—actively proliferating cells tend to be stationary, while rapidly migrating tumor cells divide more slowly, namely, the “Go-or-Grow” hypothesis (50), we also discovered activated regulons suppressing glioma cell invasion and migration (e.g., *FOXP1*) (**Figure 3B**). Furthermore, the NPC2-like tumor cells had higher metabolic activities than other tumor cellular states. Therefore, NPC2-like state cells were in the proliferating state and functioned as GBM progenitor cells, which could be a potential therapeutic target.

GBM is the most aggressive malignant brain tumor with bleak prognosis, and it contains numerous cell types. However, we still know little about which cell type may cause the poor clinical outcome of GBM patients. The gene signature of blood-derived TAMs, but not microglial TAMs, correlates with significantly inferior survival in low-grade glioma (17). Our work was the first to reveal the hypoxia-response TAMs and tumor cells in GBM strongly associated with poor prognosis. We further uncovered the chronic and acute GBM hypoxia niches that were not only related to EMT and invasion microenvironment but also involved in promoting angiogenesis. This may be one of the reasons for driving GBM progression.

TAMs are the major players in TME and are broadly divided into two phenotypes: classical M1 type involved in inflammatory response and antitumor immunity, and alternatively activated M2 type, which elicits an anti-inflammatory response and pro-tumorigenic properties (18). TAMs can shift to M2 phenotypes in response to various microenvironmental signals secreted by malignant tumor cells and stromal cells, which results in progression of tumors and poor prognosis of patients. Our work portrayed the landscape of potential ligand–receptor cross-talk pathways between GBM tumor cells and TAMs. Although MES-like tumor cells had the most ligands in promoting TAM M2 polarization and NPC-like malignant cells expressed the least relevant ligands, all GBM cellular states could participate in TAM M2-type polarization. The majority of all ten L–R pairs we identified were consistent with that reported previously in glioma and other cancers, and the remaining ones need further experimental verification. However, these findings provided new strategies to target tumor-induced M2 polarization for potential therapy.

In summary, our results revealed that NPC2-like tumor cells were in a proliferative and high energy-consumption state and could be the origin of cells in human GBM. We identified the hypoxia-response GBM cell subset, consisting of MES1-like and MES2-like tumor cells, and hypoxia-response TAMs, which were associated with worse prognosis in GBM patients through promoting invasion and angiogenesis. This study delineated the landscape of potential ligand–receptor pathways in TAM M2-like polarization, which may lead to the proposal of new strategies for the treatment of GBM.

## Data Availability Statement

The datasets presented in this study can be found in online repositories. The names of the repository/repositories and accession number(s) can be found at: https://www.ncbi.nlm.nih.gov/geo/, GSE135045.

## Ethics Statement

The studies involving human participants were reviewed and approved by the Institutional Review Board at the Nanjing Brain Hospital Affiliated to Nanjing Medical University. The patients/participants provided their written informed consent to participate in this study.

## Author Contributions

YoX, HL, HX, and RF designed the project. KY, CQ, XH, YL, LG, and YZ prepared the tumor tissue for scRNA-seq. YoX, ZW, MZ, and XT conducted the scRNA-seq experiments. YoX analyzed the data. YoX, YD, MY, GS, and YaX wrote the original draft. RF reviewed and edited the manuscript. HL provided the research funding. All authors contributed to the article and approved the submitted version.

## Funding

The research work was supported by grants from the National Natural Science Foundation of China (81972350 and 81902535), the Jiangsu Science and Education Strengthening Engineering Innovation Team Project (CXTDA2017050), the Medical Research Foundation of Jiangsu Health Commission (H2019059), and the Medical Science and Technology Development Foundation of Nanjing (ZDX16011). YoX as a visiting student researcher who received tuition support for a year from Prof. Fan’s unrestricted faculty support funds (RF).

## Conflict of Interest

RF is co-founder of IsoPlexis and Singleron Biotechnologies with financial interest.

The remaining authors declare that the research was conducted in the absence of any commercial or financial relationships that could be construed as a potential conflict of interest.

## Publisher’s Note

All claims expressed in this article are solely those of the authors and do not necessarily represent those of their affiliated organizations, or those of the publisher, the editors and the reviewers. Any product that may be evaluated in this article, or claim that may be made by its manufacturer, is not guaranteed or endorsed by the publisher.

## Supplementary Material

Supplementary Figure 1 Cell type Annotation.

Supplementary Figure 2 Correlations between GBM Cellular States and TCGA Subtypes.

Supplementary Figure 3 Developmental Trajectory of GBM Tumor Cells.

Supplementary Figure 4 Survival Analysis of GBM Cellular State Signatures.

Supplementary Figure 5 Heterogeneity of TAMs.

Supplementary Figure 6 Immunofluorescence staining for TAM-1 cluster (*CD14+ERO1A+*) in GBM.

Supplementary Figure 7 Cell-cell Communications in GBM.

Supplementary Figure 8 TAMs M2-type Polarization.

Supplementary Table 1 Patient Information.

Supplementary Table 2 Evaluation of scRNA-seq Data Quality.

Supplementary Table 3 GBM Cellular States and Cell Type Constitutions.

Supplementary Table 4 Top20 Regulons in Cellular States.

Supplementary Table 5 Top20 Regulons in TAMs.

Supplementary Table 6 DEG signatures of TAMs.

Supplementary Table 7 Antibodies used in this study.
