# Supplementary figures and images for: Single-Cell Transcriptomics Revealed Subtype-Specific Tumor Immune Microenvironments in Human Glioblastomas
Source: Front Immunol. 2022 May 20;13:914236. doi: 10.3389/fimmu.2022.914236 (PMC9163377; doi:10.3389/fimmu.2022.914236)

A

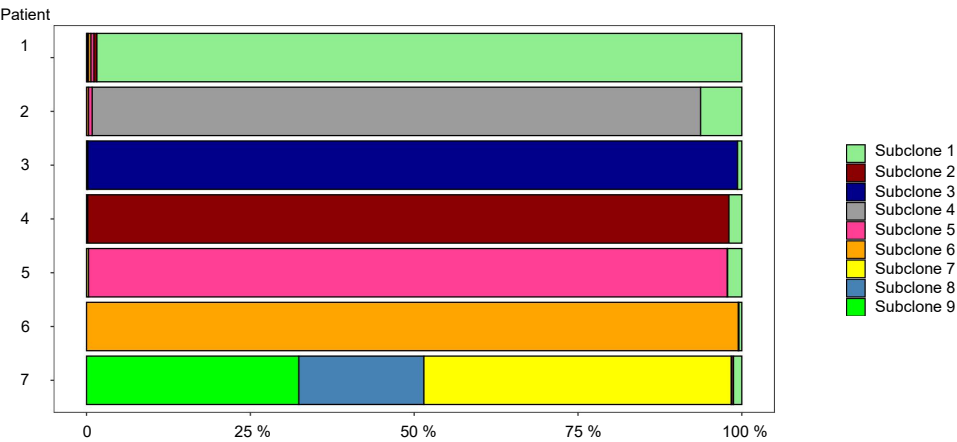

B

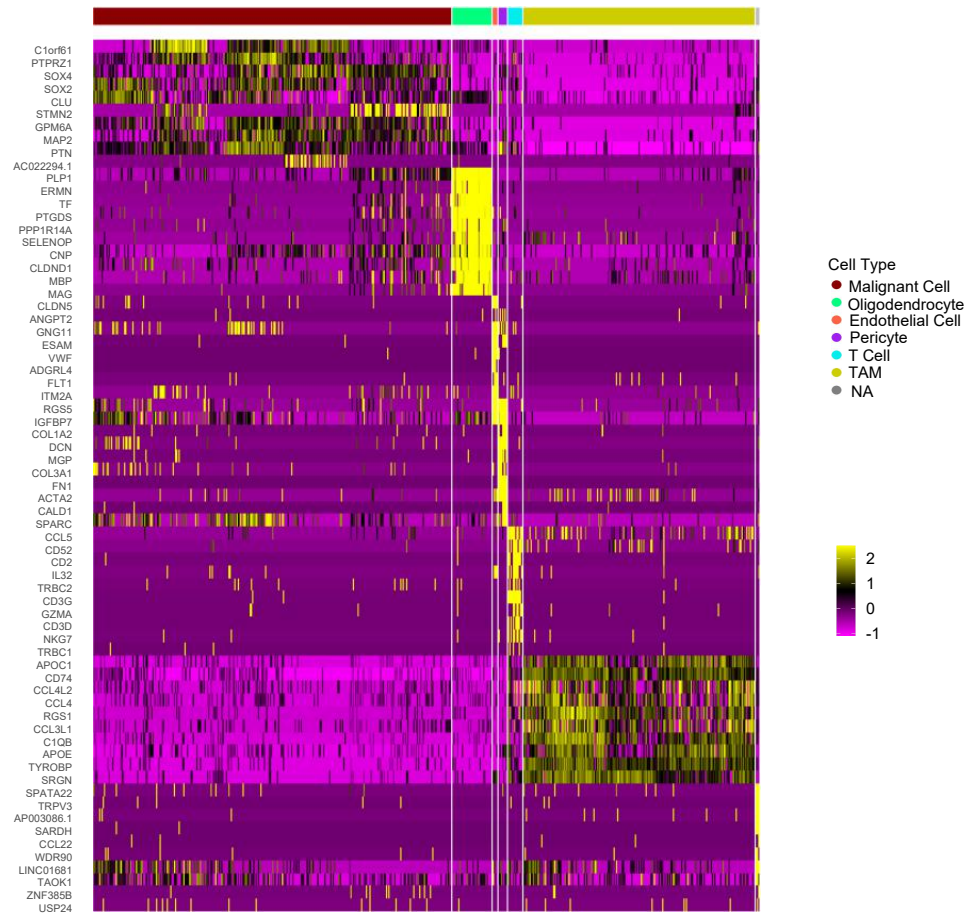

Supplement: Supplementary Figure 1 — Cell type Annotation. [file DataSheet_1.pdf]

A

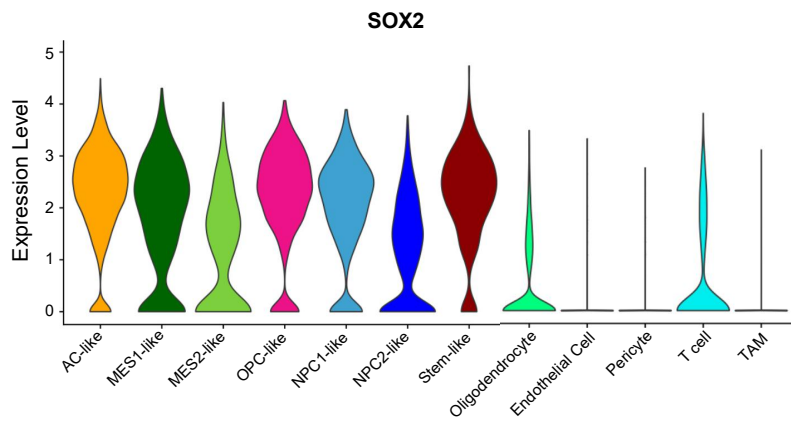

B

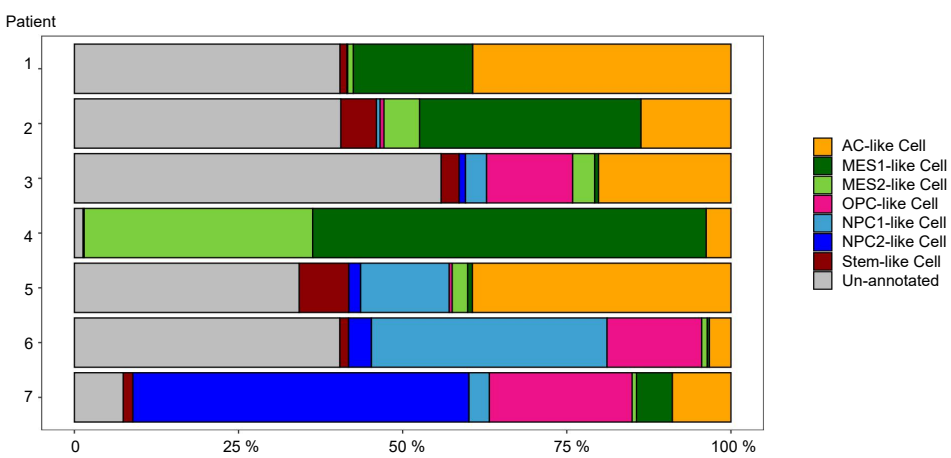

C

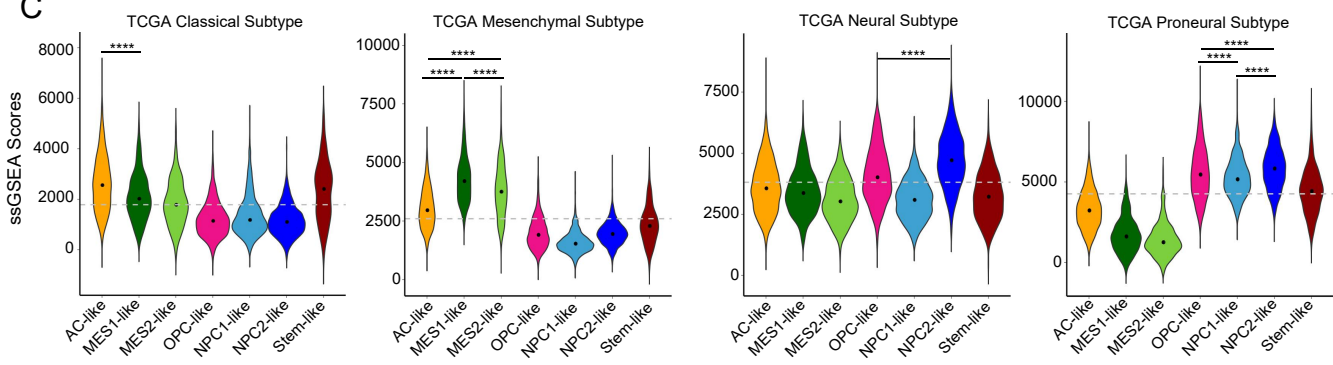

Supplement: Supplementary Figure 2 — Correlations between GBM Cellular States and TCGA Subtypes. [file DataSheet_2.pdf]

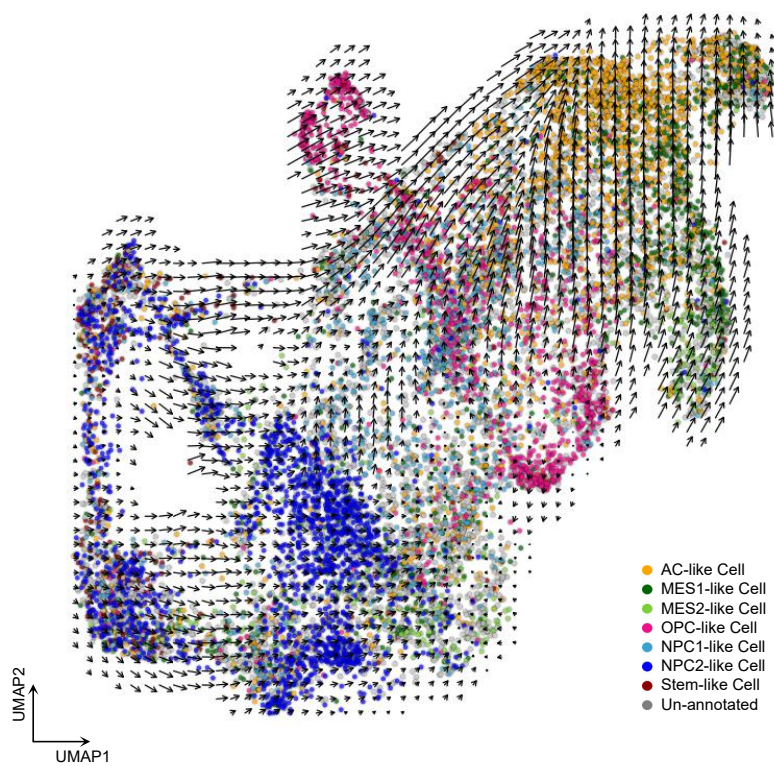

Supplement: Supplementary Figure 3 — Developmental Trajectory of GBM Tumor Cells. [file DataSheet_3.pdf]

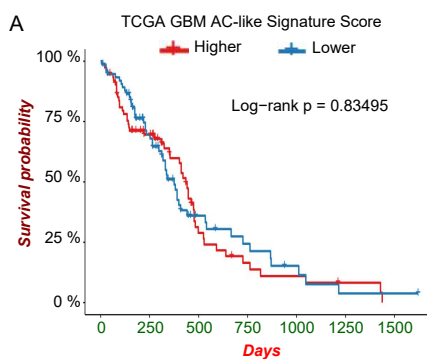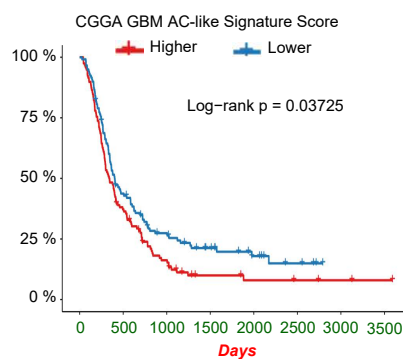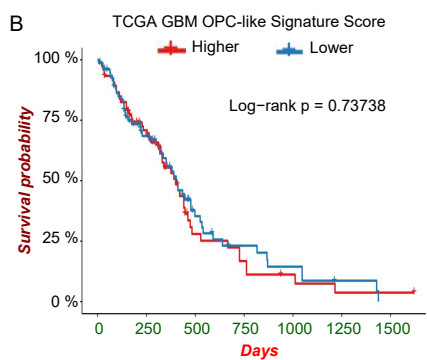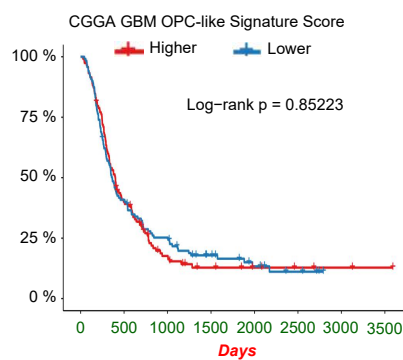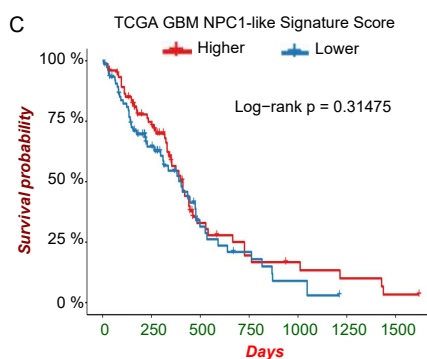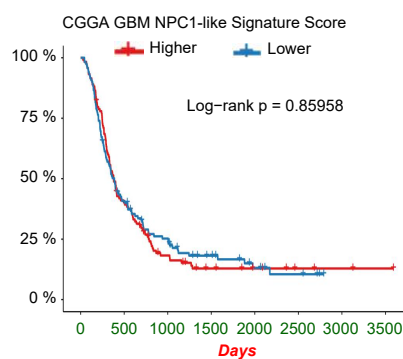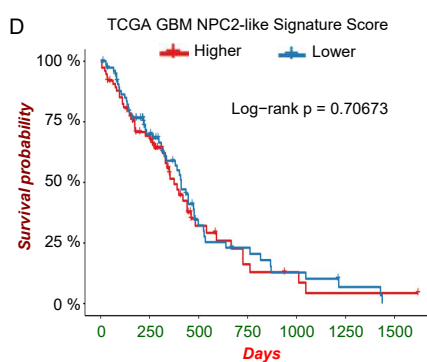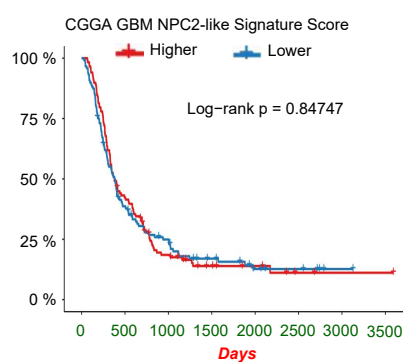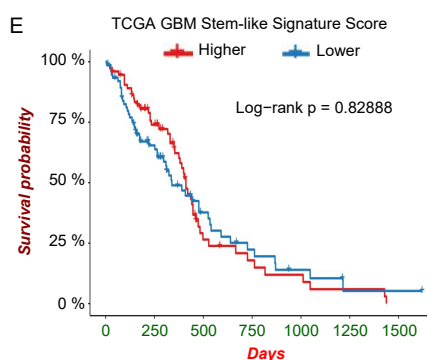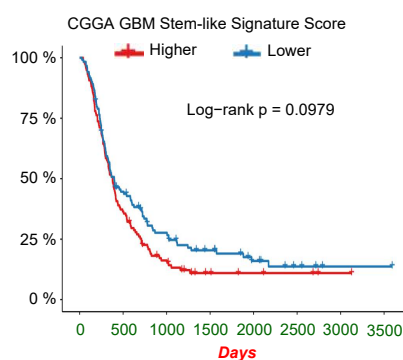

Supplement: Supplementary Figure 4 — Survival Analysis of GBM Cellular State Signatures. [file DataSheet_4.pdf]

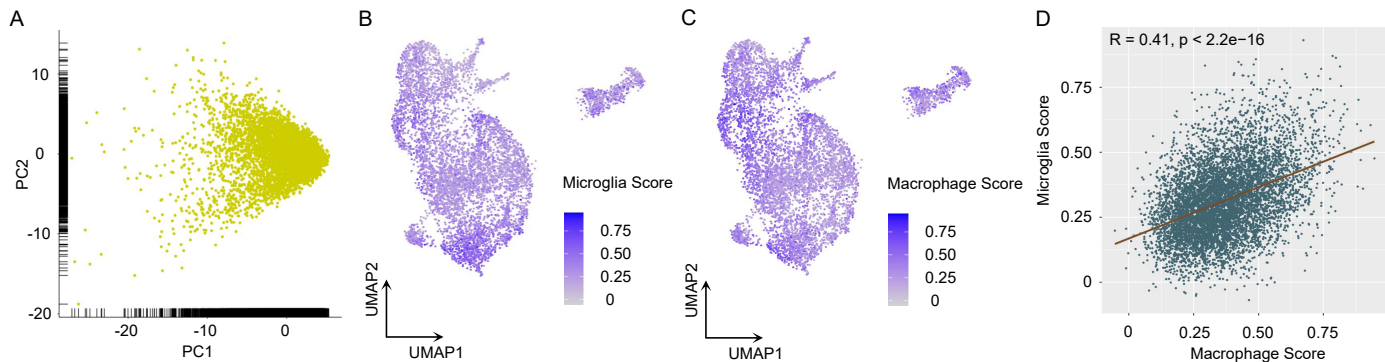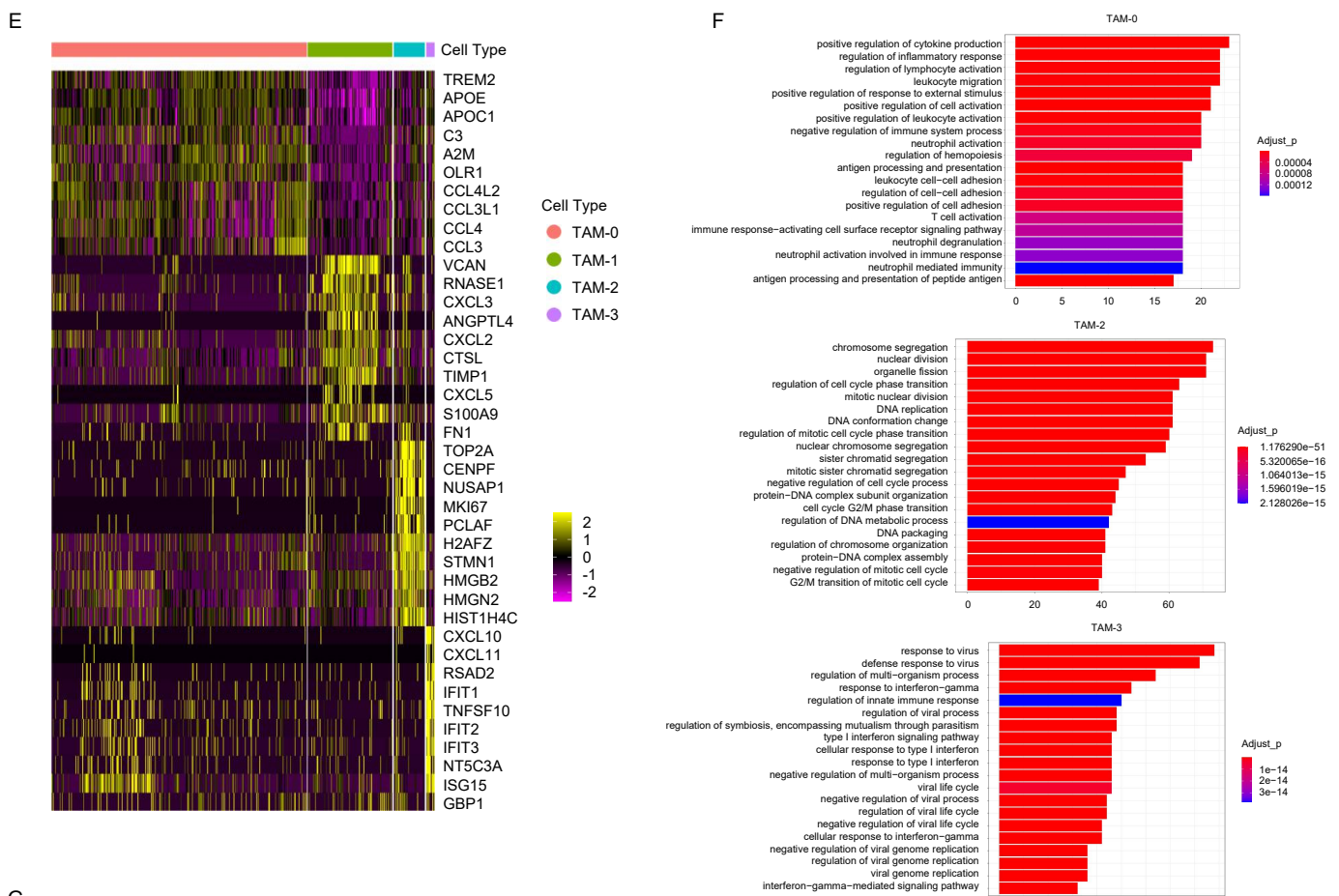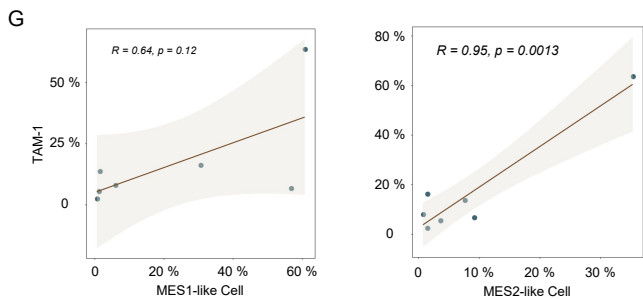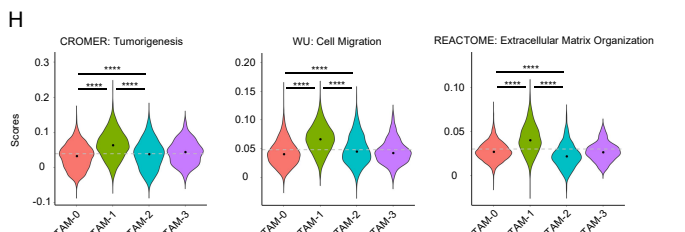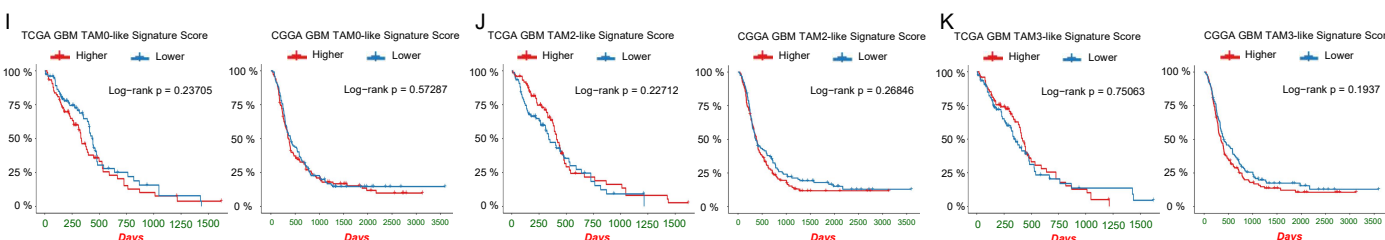

Supplement: Supplementary Figure 5 — Heterogeneity of TAMs. [file DataSheet_5.pdf]

A

CD14

ERO1A

Merge

Patient1

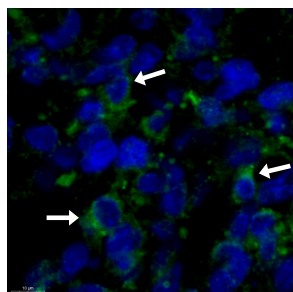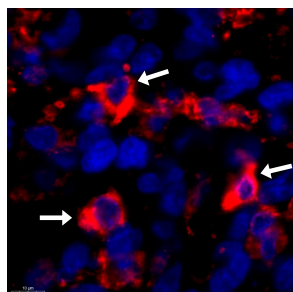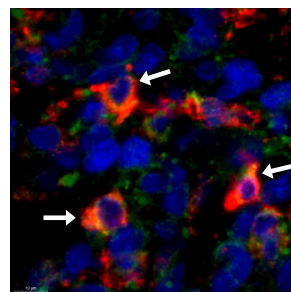

Patient2

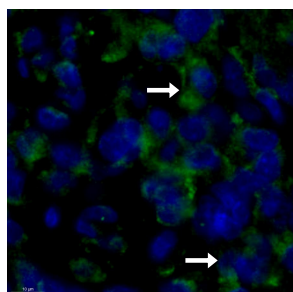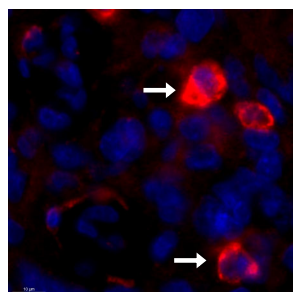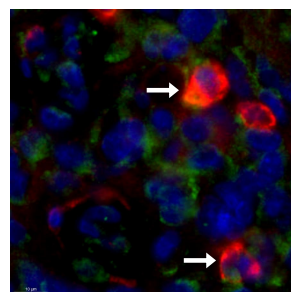

Patient4

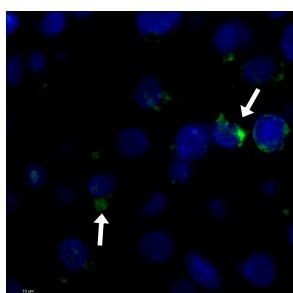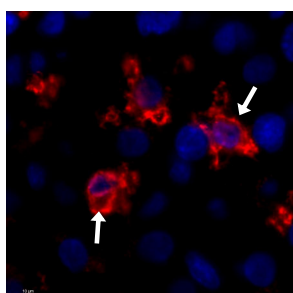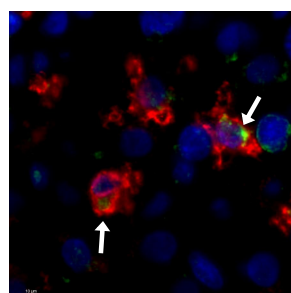

Patient5

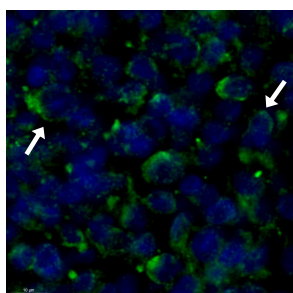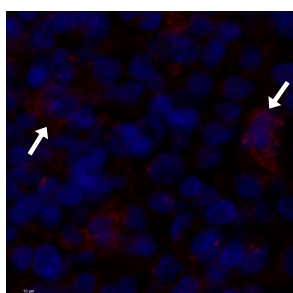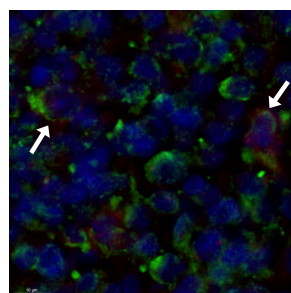

B

CD14

ERO1A

Merge

Patient3

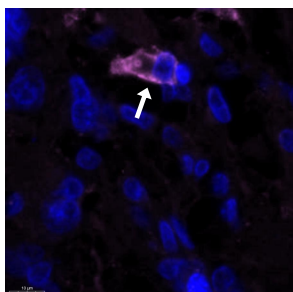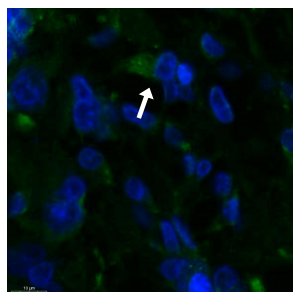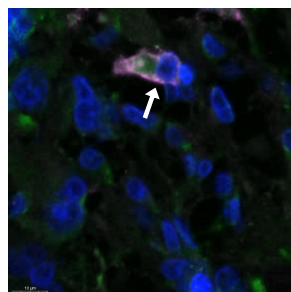

Patient7

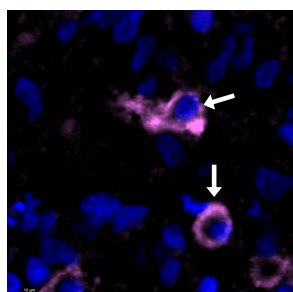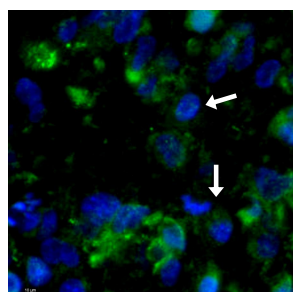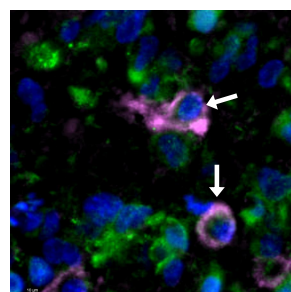

Supplement: Supplementary Figure 6 — Immunofluorescence staining for TAM-1 cluster (CD14+ERO1A+) in GBM. [file DataSheet_6.pdf]

A

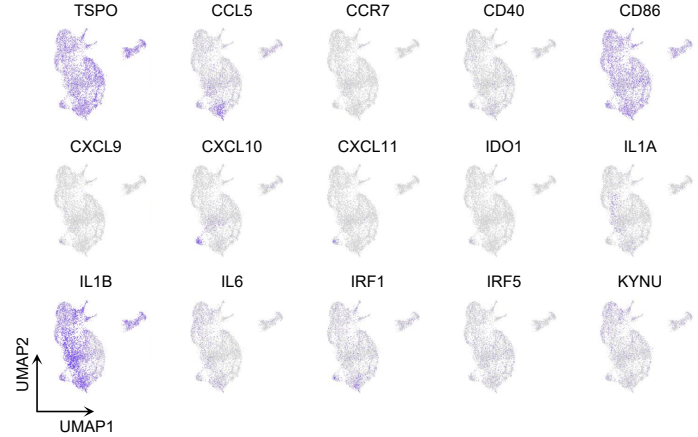

B

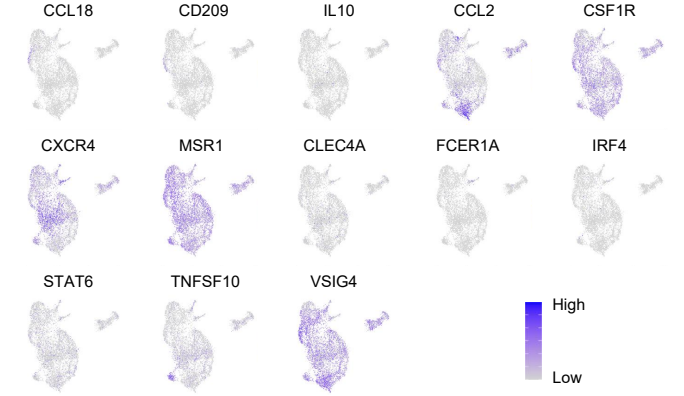

C

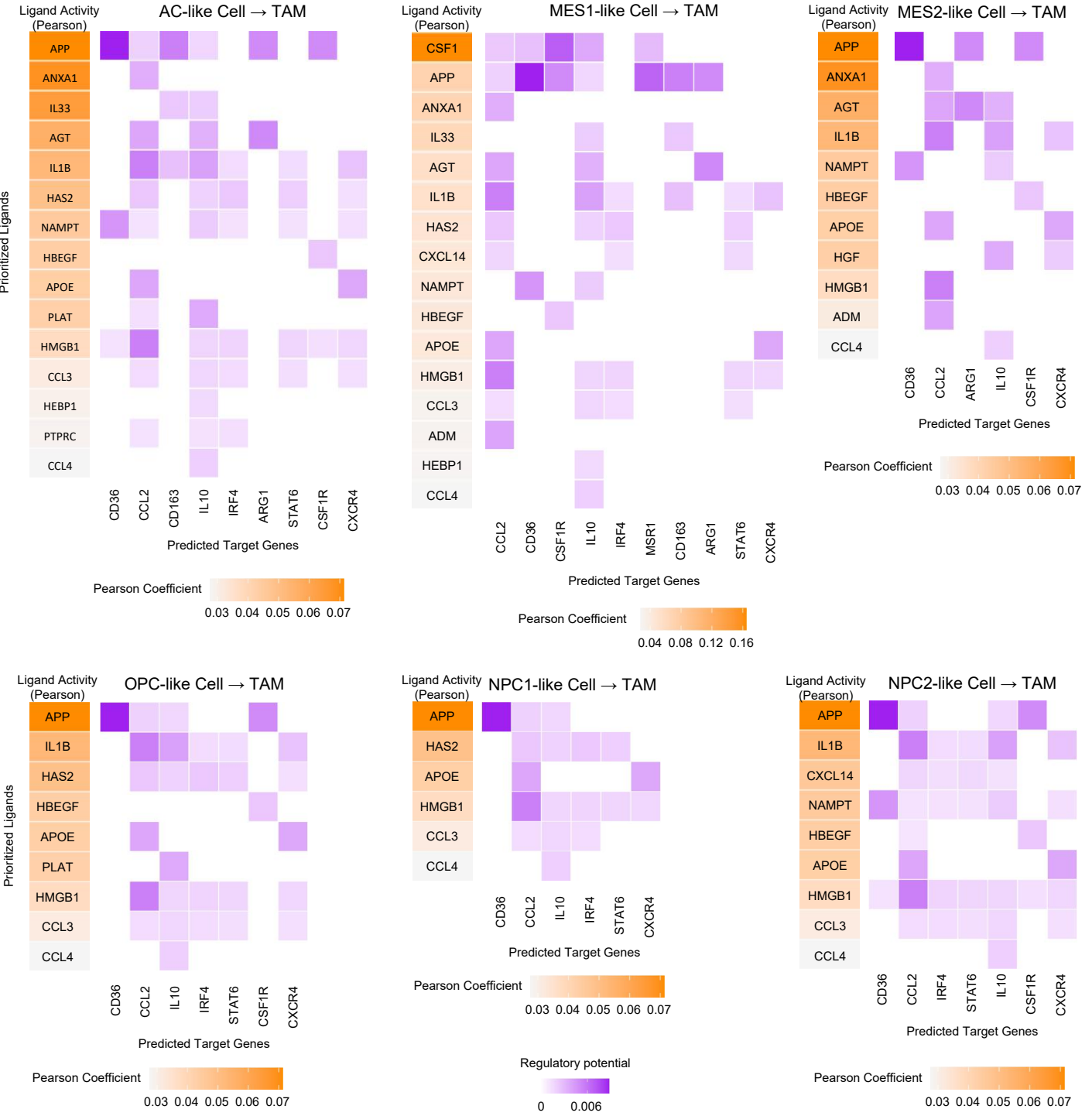

Supplement: Supplementary Figure 8 — TAMs M2-type Polarization. [file DataSheet_8.pdf]
