# Supplementary material for: Single-Cell Transcriptomics Revealed Subtype-Specific Tumor Immune Microenvironments in Human Glioblastomas
Source: Front Immunol. 2022 May 20;13:914236. doi: 10.3389/fimmu.2022.914236 (PMC9163377; doi:10.3389/fimmu.2022.914236)
Supplement: Supplementary Figure 7 — Cell-cell Communications in GBM. [file DataSheet_7.pdf]

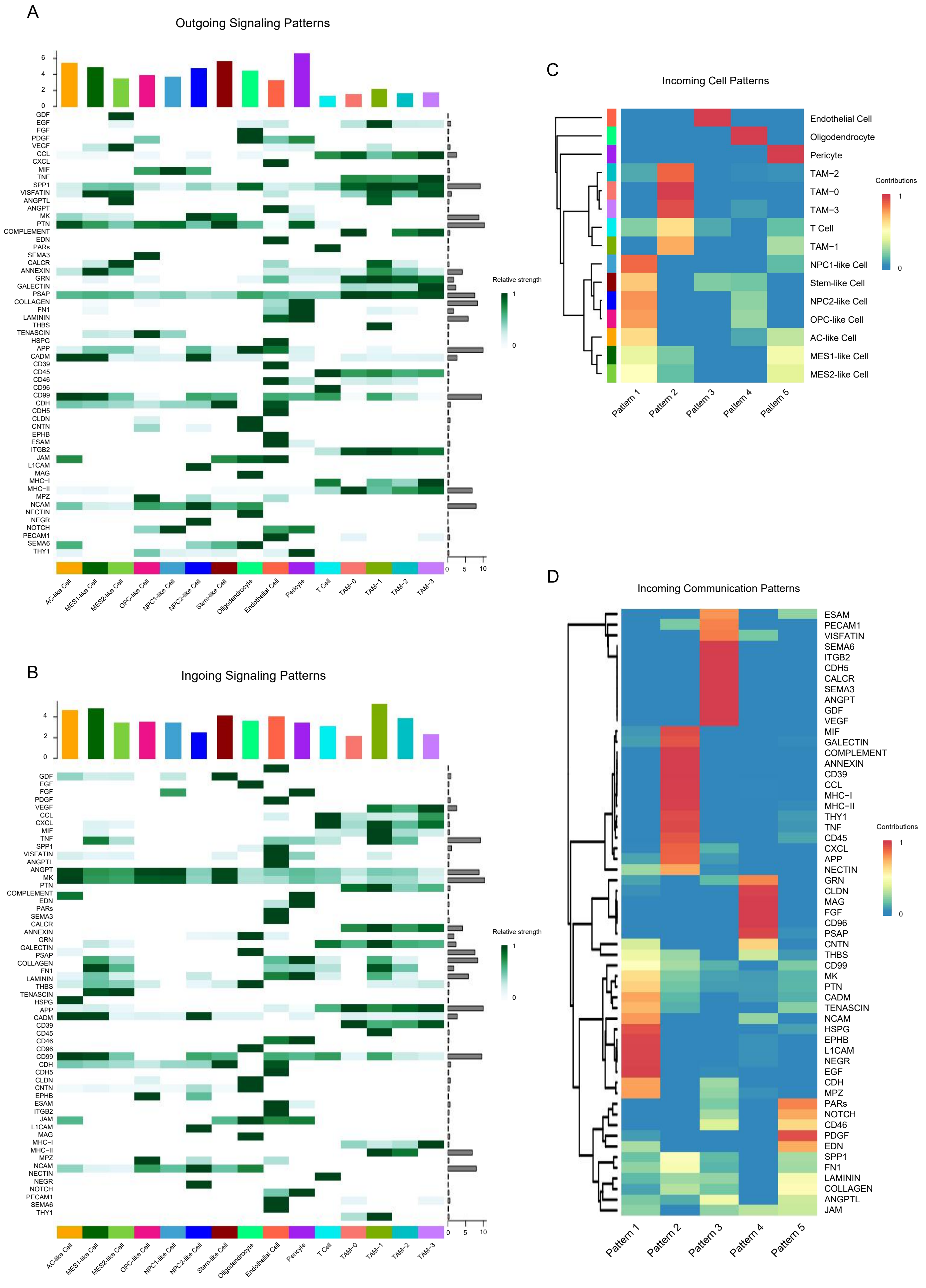

A
Outgoing Signaling Patterns
GDF
EGF
FGF
PDGF
VEGF
CCL
CXCL
MIF
TNF
SPP1
VISFATIN
ANGPTL
ANGPT
MK
PTN
COMPLEMENT
EDN
PARs
SEMA3
CALCR
ANNEXIN
GRN
GALECTIN
PSAP
COLLAGEN
FN1
LAMININ
THBS
TENASCIN
HSPG
APP
CADM
CD39
CD45
CD46
CD96
CD99
CDH
CDH5
CLDN
CNTN
EPHB
ESAM
ITGB2
JAM
L1CAM
MAG
MHC−I
MHC−II
MPZ
NCAM
NECTIN
NEGR
NOTCH
PECAM1
SEMA6
THY1
Relative strength
AC-like Cell
MES1-like Cell
MES2-like Cell
OPC-like Cell
NPC1-like Cell
NPC2-like Cell
Stem-like Cell
Oligodendrocyte
Endothelial Cell
Pericyte
T Cell
TAM−0
TAM−1
TAM−2
TAM−3
B
Ingoing Signaling Patterns
Relative strength
C
Incoming Cell Patterns
Endothelial Cell
Oligodendrocyte
Pericyte
TAM−2
TAM−0
TAM−3
T Cell
TAM−1
NPC1-like Cell
Stem-like Cell
NPC2-like Cell
OPC-like Cell
AC-like Cell
MES1-like Cell
MES2-like Cell
Contributions
Pattern 1
Pattern 2
Pattern 3
Pattern 4
Pattern 5
D
Incoming Communication Patterns
ESAM
PECAM1
VISFATIN
SEMA6
ITGB2
CDH5
CALCR
SEMA3
ANGPT
GDF
VEGF
MIF
GALECTIN
COMPLEMENT
ANNEXIN
CD39
CCL
MHC−I
MHC−II
THY1
TNF
CD45
CXCL
APP
NECTIN
GRN
CLDN
MAG
FGF
CD96
PSAP
CNTN
THBS
CD99
MK
PTN
CADM
TENASCIN
NCAM
HSPG
EPHB
L1CAM
NEGR
EGF
CDH
MPZ
PARs
NOTCH
CD46
PDGF
EDN
SPP1
FN1
LAMININ
COLLAGEN
ANGPTL
JAM
Contributions
